# Comprehensive analysis of alternative splicing across multiple transcriptomic cohorts reveals prognostic signatures in prostate cancer

**DOI:** 10.1186/s40246-023-00545-w

**Published:** 2023-11-03

**Authors:** Zhuofan Mou, Jack Spencer, John S. McGrath, Lorna W. Harries

**Affiliations:** 1 Clinical and Biomedical Sciences, Department of Clinical and Biomedical Sciences, University of Exeter Medical School, Faculty of Health and Life Sciences, University of Exeter, Barrack Road, Exeter, EX2 5DW UK; 2 Translational Research Exchange at Exeter, Living Systems Institute, University of Exeter, Exeter, UK; 3 Royal Devon University Healthcare NHS Foundation Trust, Barrack Road, Exeter, EX2 5DW UK

**Keywords:** Alternative splicing, Microarray, RNA-seq, Prognosis, Prostate cancer

## Abstract

**Background:**

Alternative splicing (AS) plays a crucial role in transcriptomic diversity and is a hallmark of cancer that profoundly influences the development and progression of prostate cancer (PCa), a prevalent and potentially life-limiting cancer among men. Accumulating evidence has highlighted the association between AS dysregulation and the onset and progression of PCa. However, a comprehensive and integrative analysis of AS profiles at the event level, utilising data from multiple high-throughput cohorts and evaluating the prognosis of PCa progression, remains lacking and calls for thorough exploration.

**Results:**

We identified a differentially expressed retained intron event in *ZWINT* across three distinct cohorts, encompassing an original array-based dataset profiled by us previously and two RNA sequencing (RNA-seq) datasets. Subsequent in-depth analyses of these RNA-seq datasets revealed  141 altered events, of which 21 demonstrated a significant association with patients’ biochemical recurrence-free survival (BCRFS). We formulated an AS event-based prognostic signature, capturing six pivotal events in genes *CYP4F12*, *NFATC4*, *PIGO*, *CYP3A5*, *ALS2CL*, and *FXYD3*. This signature effectively differentiated  high-risk patients diagnosed with PCa, who experienced shorter BCRFS, from their low-risk counterparts. Notably, the signature's predictive power surpassed traditional clinicopathological markers in forecasting 5-year BCRFS, demonstrating robust performance in both internal and external validation sets. Lastly, we constructed a novel nomogram that integrates patients’ Gleason scores with pathological tumour stages, demonstrating improved prognostication of BCRFS.

**Conclusions:**

Prediction of clinical progression remains elusive in PCa. This research uncovers novel splicing events associated with BCRFS, augmenting existing prognostic tools, thus potentially refining clinical decision-making.

**Supplementary Information:**

The online version contains supplementary material available at 10.1186/s40246-023-00545-w.

## Background

Prostate cancer (PCa) is the commonest cancer affecting the male reproductive system and stands as the second leading cause of male mortality globally, with men over 65 years of age being at elevated risk [1, 2]. Previous research has shown that RNA alternative splicing (AS), a fundamental biological process that results in the generation of diverse mRNA isoforms encoding distinct transcripts [3] plays a pivotal role in PCa progression and aggressiveness [4–6]. Dysregulation or malfunction of AS is associated with cellular dysfunction and the pathogenesis of various diseases, including cardiovascular disease [7], neurological disorders [8, 9], and cancers [10–12]. Mutations or alterations in the expression of splicing factors can facilitate the production of cancer-promoting splicing isoforms, thereby granting growth or survival advantages to tumour cells [10]. Consequently, aberrant AS has been proposed as a hallmark of cancer [13]. Harnessing the power of genome-wide transcriptome approaches will uncover the full potential of AS events as PCa markers. These approaches will facilitate identification of individuals at greater risk of progression or recurrence and may allow clinicians to devise personalised treatment plans that optimise efficacy while minimising side effects. Furthermore, investigating the associations between cancer-specific splicing events and disease features may indicate future prognostic biomarkers and therapeutic targets, ultimately offering the potential for improved outcomes for patients diagnosed with PCa [14–16].

AS significantly contributes to transcriptomic and proteomic diversification in eukaryotes, with approximately 95% of human genes undergoing AS to produce proteins exhibiting distinct functions [17]. Grasping the intricate function of AS may illuminate the enigmatic pathology of PCa. AS events can be classified into five primary categories: skipped exon (SE), alternative 5′ splice site (A5SS), alternative 3′ splice site (A3SS), mutually exclusive exons (MXE), and retained intron (RI). Each of these has the potential to have profound effects on the nature, abundance, or stability of the resultant transcripts and consequently, on the functionality of the gene product. The advent of ultra-high density microarray technologies and high-throughput RNA sequencing (RNA-seq) techniques has enabled researchers to apply bioinformatics methodologies to large transcriptome-wide data to identify expression or splicing changes that can inform on disease parameters. Tools developed for AS analysis can be categorised into three types depending on their mathematical background, including event-based, exon-based, and isoform-based approaches [18]. Tools such as rMATS [19], SpliceSeq [20], and EventPointer [21] are event-based and can be employed to query the association of specific AS events with clinical parameter such as disease status or risk of recurrence.

In this study, we aimed to comprehensively explore the AS landscape of PCa utilising advanced AS analytical tools. We thoroughly examined our previous array-based PCa cohort alongside three independent high-throughput transcriptomic PCa datasets at the event-level of AS. This approach was designed to counter potential biases from single studies and to identify shared patterns of differentially expressed alternative splicing events, termed DEAS events, across the respective datasets. Subsequently, we sought to pinpoint a robust set of prognostic events and define a minimal event-based signature associated with biochemical recurrence-free survival (BCRFS) in patients diagnosed with PCa. Biochemical recurrence is defined as a rise in the blood level of prostate-specific antigen (PSA) in patients diagnosed with PCa after treatment with surgery or radiation. Additionally, we constructed a correlation network between aberrantly altered splicing factors and the prognostic events to uncover potential upstream splicing regulators. We developed an AS event signature capable of predicting 3-, 5-, and 8-year BCRFS in patients diagnosed with PCa, validated both internally and externally. Furthermore, we established a nomogram incorporating the AS event signature and clinicopathological factors to predict BCRFS at the same time intervals. Both the nomogram and the AS signature outperformed the Gleason score in BCRFS prediction, indicating their potential utility in PCa clinical management. Given the critical role of AS mechanisms in PCa, our findings have identified potential prognostic AS event biomarkers which may aid clinicians when designed treatment or follow-up regimens. Furthermore, the results here may suggest novel potential targets for future PCa therapeutics designed to manipulate splicing decisions.

## Materials and methods

### Datasets acquisition

In this work, four independent PCa datasets were analysed and are summarised in Table 1.

**Table 1:** Overview of the four study cohorts used in this study

Dataset	Title of study	Sample description	Platform	Total number of samples	Number of tumour samples	Number of normal samples	BCR events	Tool used	References
Clariom D	Gene expression analysis reveals a 5-gene signature for progression-free survival in prostate cancer	Benign and matched prostate cancer tissue was obtained using radical prostatectomy specimens from nine patients	Affymetrix Clariom D Human micro-array	18	9	9	NA	EventPointer	Romero et al. [21]
TCGA-PRAD	Prostate Adenocarcinoma (The Cancer Genome Atlas)	Surgical resection biospecimens were collected from patients diagnosed with prostate adenocarcinoma, and had not received prior treatment for their disease	Illumina HiSeq 2000	541	489	52	67	SpliceSeq	Ryan et al. [20]
PRJEB2449	RNA-seq analysis of prostate cancer in the Chinese population identifies recurrent gene fusions, cancer-associated long noncoding RNAs and aberrant alternative splicings	Prostate cancer and matched adjacent normal tissues were obtained from patients diagnosed with prostate cancer	Illumina HiSeq 2000	28	14	14	NA	rMATS	Shen et al. [19]
GSE107299	The Proteogenomic Landscape of Curable Prostate Cancer	Prostate tumours obtained from patients underwent either image-guided radiotherapy or radical prostatectomy	Affymetrix Human Transcriptome Array 2.0 (HTA 2.0) (Batch 2)	61	61	NA	17	EventPointer	Romero et al. [21]

*TCGA* The Cancer Genome Atlas, *PRAD* prostate adenocarcinoma, *BCR* biochemical recurrence-free, *NA* no samples available

**Fig. 1:**
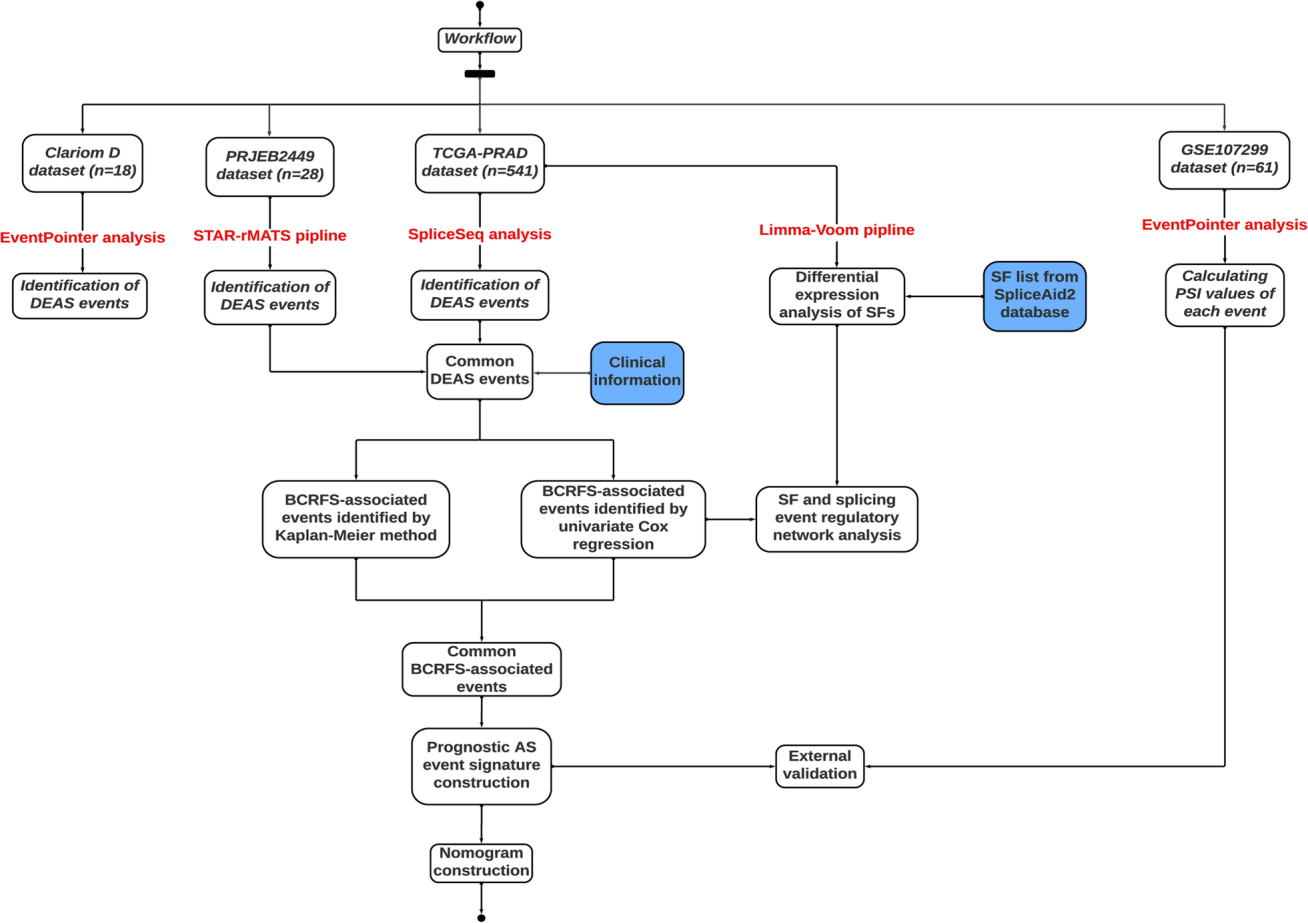
Flowchart illustrating workflow. The flowchart demonstrates the major steps employed in the study. *DEAS* differentially expressed alternative splicing, *SF* splicing factor, *BCRFS* biochemical recurrence-free survival, *TCGA* The Cancer Genome Atlas, *PRAD* prostate adenocarcinoma

The first dataset, referred to as Clariom D, is a series of nine paired samples consisting of benign and malignant tissue from the same patient. Transcriptomes were produced using the ultra-high density the Clariom D Pico GeneChip Whole Transcriptome (WT) platform (Thermo Fisher, Waltham, MA, USA). Analysis, patient anthropometrics and clinical parameters for this patient group have been described in our previous paper [22]. The second dataset, referred to as TCGA-PRAD (https://portal.gdc.cancer.gov/projects/TCGA-PRAD), involves 541 normal and tumour prostate samples. RNA-seq counts, clinical, pathological, and survival information for patients diagnosed with PCa were obtained from the Genomics Data Commons (GDC) [23] using the R package TCGAbiolinks [24]. The third dataset, generated by Illumina HiSeq 2000, consists of 14 PCa tumour and 14 matched normal samples [25] and was obtained from the European Nucleotide Archive (ENA) [26] using fastq-dl [27] under the accession number PRJEB2449. The fourth and final dataset includes 61 tumour samples [28] and only batch 2 samples were selected, which had been profiled with the Affymetrix Human Transcriptome Array 2.0 (HTA 2.0), for this study. Raw CEL data (i.e. probe intensities) and corresponding survival information were downloaded from the Gene Expression Omnibus (GEO) [29] under the accession number GSE107299 (date accessed: 30/09/2022) and PCaDB (http://bioinfo.jialab-ucr.org/PCaDB/; date accessed: 11/05/2023), respectively. In this study, we used BCRFS as our survival endpoint. Patients with BCRFS less than one month were excluded. The design of this study is illustrated as a flowchart in Fig. 1.

### Identification of DEAS events in Clariom D array dataset

The raw array CEL data of the Clariom D dataset were pre-processed using the R package EventPointer under the *aroma.affymetrix* framework [30]. We conducted differential splicing event analysis between the nine malignant and nine matched benign prostate samples, using the R package EventPointer and annotated the results using genome reference GRCh38. EventPointer computes percent-spliced-in (PSI) to quantify each AS event. For discovery, significant DEAS events associated with PCa were screened with unadjusted *p* < 0·01 and |ΔPSI|> 0·1. To explore the relevance of the parent genes of the DEAS events to cancer, we used CancerMine, a literature-based database, to categorise them into key drivers, oncogenes, and/or tumour suppressors [31].

### Identification of DEAS events in TCGA-PRAD dataset

The raw counts of the TCGA-PRAD cohort were pre-processed using the R package edgeR [32], and differential gene expression analysis was performed using Limma–Voom pipeline [33, 34]. The PSI values, which represent the ratio between reads including or excluding exons, were retrieved from the TCGA SpliceSeq database for the TCGA-PRAD cohort (https://bioinformatics.mdanderson.org/TCGASpliceSeq/PSIdownload.jsp; date accessed: 09/05/2022). To ensure a reliable dataset of splicing events, we applied stringent thresholds to exclude (a) splicing events for which the PSI values were missing or not expressed in more than 25% of the samples and (b) samples with missing/NA events greater than 30%. The remaining missing events were imputed using K-nearest neighbours (KNN). We retained AS events with average PSI > 0·05 and standard deviation > 0·01 for downstream analysis. Each splicing event was assigned a unique identifier consisting of the splicing type, gene symbol, and ID number to facilitate event identification and ensure accuracy. For example, an event ID 'ZWINT|11811|RI' consists of a parent gene symbol 'ZWINT' and a unique ID number '11811' assigned to the 'RI' event type. We identified DEAS events between PCa tumour and normal samples in the TCGA-PRAD dataset using the R package limma [33] based on the PSI values. Significant DEAS events were screened using BH FDR-adjusted *p* < 0·05.

### Identification of DEAS events in PRJEB2449 dataset

The raw RNA-seq reads in the PRJEB2449 dataset were analysed to identify DEAS events between 14 PCa tumour and 14 matched normal samples. Quality assessment was performed using FastQC [35] and MultiQC [36]. Low-quality reads were trimmed to have a minimum length greater than 20 base pairs using TrimGalore [37]. Human genome sequencing reference and annotation files were downloaded from the Ensembl database (version: GRCh38/hg38, release 108) and indexed using Spliced Transcripts Alignment to a Reference (STAR) [38]. Reads from each sample were mapped to the genome reference hg38 and quantified using STAR. Subsequently, we employed rMATS (v4.0.2) to conduct a pairwise statistical analysis between tumour versus normal group comparison and identify significant DEAS events with BH FDR-adjusted *p* < 0·05. To effectively illustrate the diversity and prevalence of the splicing patterns, we used sashimi plots to provide an intuitive visualisation of RNA-seq data and splicing junctions. The analysis of this dataset was conducted in a Linux environment, utilising remote ISCA High-Performance Computing (HPC) clusters supported by the University of Exeter.

### Concordance and validation of DEAS events across datasets

To identify overlapping DEAS events across different cohorts, we used the UCSC LiftOver tool (https://genome.ucsc.edu/cgi-bin/hgLiftOver) to convert the genomic coordinates of each event from reference assembly GRCh38 to GRCh37, or vice versa. Finally, we confirmed the overlapping DEAS events by comparing the genomic coordinates of each event along with the UCSC Genome Browser. This ensured that the same event was identified in each cohort and allowed us to perform downstream analyses with confidence.

### Identification of prognostic AS events and construction of potential splicing regulatory network

We extracted overlapping DEAS events between the TCGA-PRAD and PRJEB2449 cohorts and performed univariate Cox regression analysis using the TCGA-PRAD set to obtain events that were associated with BCRFS (with *p* < 0·05). To identify putative regulators of the BCRFS-associated DEAS events, an initial list of tissue-specific and experimentally validated splicing factors were retrieved from the SpliceAid2 database [39] (www.introni.it/spliceaid.html; date accessed: 11/05/2023). Differentially expressed splicing factors were identified using the TCGA-PRAD RNA-seq data and Limma-Voom pipeline with BH FDR-adjusted *p* < 0·05. To explore potential upstream regulators of the prognostic AS events, Pearson correlation network analysis was conducted between the expression of dysregulated splicing factors and PSI of BCRFS-associated events. Correlation significance was set at *p* < 0·01, and the network was visualised by the Cytoscape software.

### Construction and validation of prognostic AS event model for BCRFS of PCa patients

We used the overlapping DEAS events from the two RNA-seq studies to determine their prognostic significance and establish an AS event signature model for predicting BCRFS of patients diagnosed with PCa. The TCGA-PRAD dataset was randomly divided into training (*n* = 289; 70%) and testing (*n* = 123; 30%) sets. The training set served to construct the prognostic signature, which was then evaluated in the testing set and entire dataset. Association of each overlapping event with BCRFS was evaluated via univariate Cox regression and Kaplan–Meier (KM) analysis. LASSO regression was performed on the prognostic events to minimise the residual sum of squares plus a penalty term, and thus prevent overfitting of the model. This was performed using the R package glmnet [40]. Optimal events identified were then used to construct a multivariate Cox proportional hazards model with a bidirectional stepwise variable selection, using the R package survival [41]. For each patient, the risk score was calculated based on the weighted linear combination of the event coefficient derived from the multivariate Cox regression analysis and the corresponding PSI value. Patients diagnosed with PCa were classified into high-risk and low-risk groups based on the risk scores, using the R package survminer  [42]. We used the KM method to assess if patients in the high-risk group was associated with worse survival. The prognostic model was validated on the TCGA-PRAD testing set, the entire set, and on an external dataset, GSE107299. We pre-processed the GSE107299 dataset and calculated the PSI value of each AS event using the R package EventPointer. Model efficiency in predicting 3-, 5-, and 8-year BCRFS was assessed using time-dependent receiver operating characteristic (ROC) analysis, using the R package survivalROC [43]. The area under the ROC curve (AUC) and the Harrell’s concordance index (C-index) were used to evaluate the model performance.

### Clinical significance and nomogram construction

Univariate and multivariate Cox regression analyses were conducted to evaluate the association between the event signature risk scores and BCRFS. In addition, we also took into account the widely accepted clinicopathological parameters in PCa. These parameters include patient age at diagnosis (< 60 vs. ≥ 60), Gleason score (≤ 7 vs. > 7), pathological T stage (T2 vs. T3–T4), and pathological N stage (N0 vs. N1). We employed the Student’s t-test to investigate the differences in of risk scores between the two clinically delineated patient groups, considering a *p* < 0·05 as statistically significant. A nomogram was constructed for predicting BCRFS at 3-, 5-, and 8-year intervals using the parameters that proved statistical significance (*p* < 0·05) from the multivariate Cox regression analysis conducted on the TCGA-PRAD training set. The predictive performance of the nomogram was further validated internally using the TCGA-PRAD dataset using the ROC analysis and the C-index.

## Results

### Identification of PCa-associated aberrant AS events in Clariom D array dataset

**Fig. 2:**
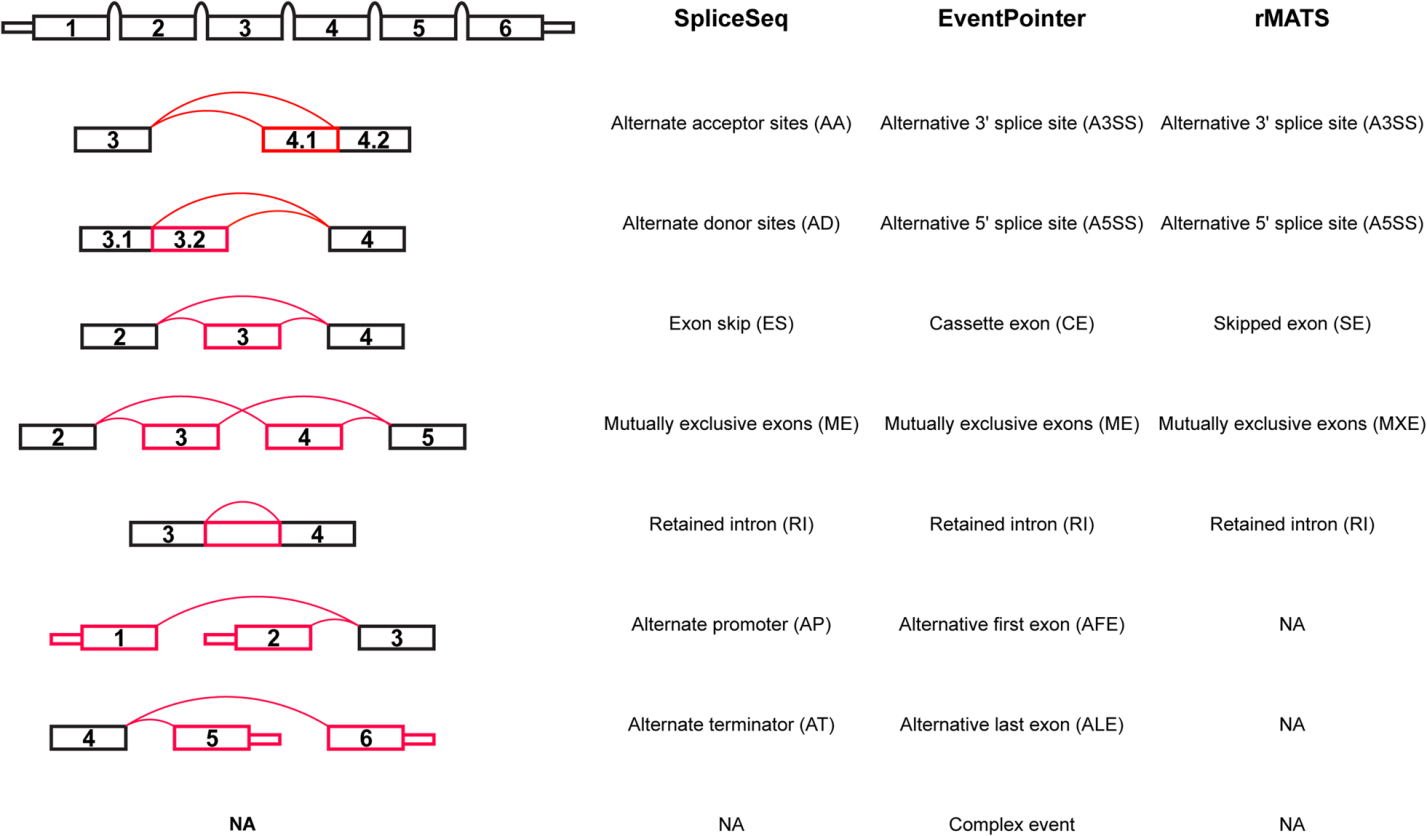
Splice model illustrating event types identified by SpliceSeq, EventPointer, and rMTAS. Each box is individually annotated with an exon number. Splice junctions are represented by red curves, while red boxes signify alternatively spliced exons. *NA* not applicable

Event types that can be detected by each AS tool are illustrated and summarised in Fig. 2.

**Fig. 3:**
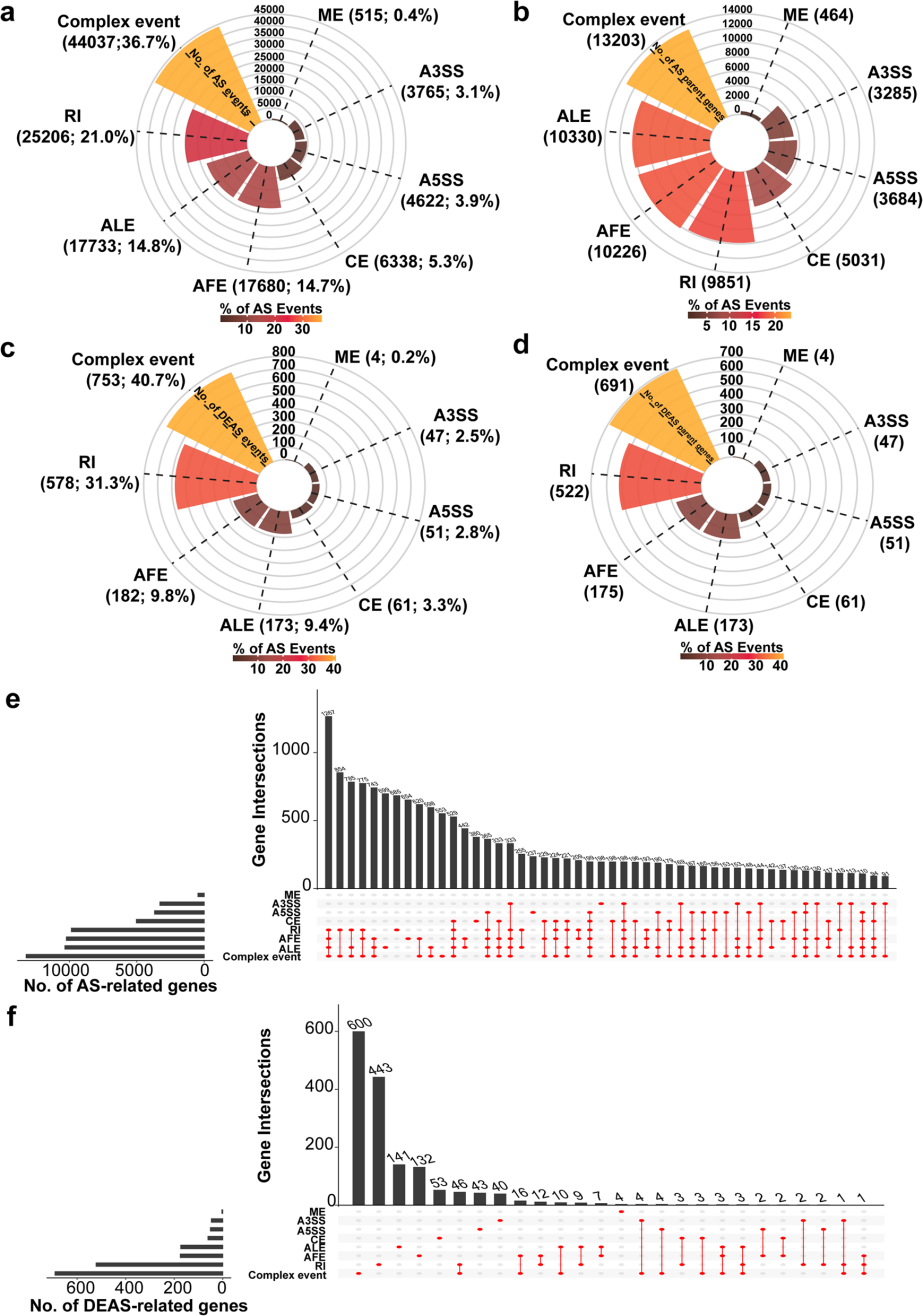
Detected AS and DEAS Events in malignant vs. benign prostate samples: Clariom D cohort. **a** Circle plot shows the count and proportion of detected alternative splicing (AS) events, while **c** presents the differentially expressed alternative splicing (DEAS) events, broken down by event type. Circle plots **b** and **d** represent the number of parent genes implicated in detected AS and DEAS events, respectively, per event type. **e** UpSet plot elucidates the detected AS event parent genes, indicating the number of genes engaged in distinct event types (illustrated by horizontal bars) and their involvement in various event type combinations (represented by vertical bars and connected red dots). The UpSet plot **f** does the same for DEAS event parent genes. *RI* retained intron, *A3SS* alternative 3′ splice site, *A5SS* alternative 5′ splice site, *CE* cassette exon, *ME* mutually exclusive exons, *AFE* alternative first exon, *ALE* alternative last exon

EventPointer differentiates events into eight categories, including mutually exclusive exons (ME), alternative 3′ splice site (A3SS), alternative 5′ splice site (A5SS), cassette exon (CE), alternative last exon (ALE), alternative first exon (AFE), retained intron (RI) and complex event (i.e. none of the standard categories above). Of the 171,994 found AS events, 119,896 were annotated with a gene name. These comprise 515 MEs in 464 genes, 3765 A3SSs in 3285 genes, 4622 A5SSs in 3684 genes, 6338 CEs in 5031 genes, 17,733 ALEs in 10,330 genes, 17,680 AFEs in 10,226 genes, 25,206 RIs in 9851 genes, and 44,037 complex events in 13,203 genes. The number of events and the associated parent genes detected in each event type are summarised in Fig. 3a, b, respectively. Among these, 1849 annotated events deriving from 1586 genes demonstrated significant differential expression in relation to PCa. These include 4 MEs in 4 genes, 47 A3SSs in 47 genes, 51 A5SSs in 51 genes, 61 CEs in 61 genes, 173 ALEs in 173 genes, 182 AFEs in 175 genes, 578 RIs in 522 genes, and 753 Complex Events in 691 genes (Fig. 3c, d; Additional file 1: Table S1). The resulting events highlighted some genes exhibited multiple AS events, a prevalent mechanism for generating protein diversity that may contribute to malignant tumour formation and progression (Fig. 3e, f). Differentially regulated events and the top 10 up-regulated and down-regulated AS events, ranked in ascending p-value, are listed in Table 2. The CancerMine database indicated that around 32% of the DSGs have been previously identified or cited as key biomarkers with different roles, including 138 drivers, 393 oncogenes, and 237 tumour suppressors in different cancer types. Among these genes, the two most frequently cited genes were *TP53* (1837 overall; 49 citations revealing as a tumour suppressor (35), driver (9), and oncogene (5) in PCa) and *MET* (417 overall; 4 citations playing as an oncogene in PCa) (Additional file 2: Table S2).

**Table 2:** Top 10 up-regulated and down-regulated alternative splicing events in the Clariom D dataset

ProbeID	Gene name	Event type	Genomic position	Splicing *Z* value	Splicing *p* value	Delta PSI
*Top10 up-regulated events*						
TC1500008343.hg_3	UNC45A	Alternative Last Exon	15:90931052–90933977	− 5.30043915	1.16E−07	0.200665185
TC0600011184.hg_1	HIST1H2BJ	Complex Event	6:27126462–27132525	− 5.059966554	4.19E−07	0.152888644
TC1700011923.hg_6	CANT1	Complex Event	17:78997774–78998148	− 4.997983001	5.79E−07	0.2510289
TC0100018146.hg_1	ZNF692	Complex Event	1:248850516–248853937	− 4.820815714	1.43E−06	0.106067575
TC0100008145.hg_1	TSPAN1	Complex Event	1:46175409–46181100	− 4.75097649	2.02E−06	0.258356425
TC0400007928.hg_7	FRAS1	Cassette Exon	4:78419063–78424388	− 4.661850479	3.13E−06	0.13807046
TC1100013040.hg_13	TM7SF2	Complex Event	11:65115394–65115893	− 4.572538539	4.82E−06	0.121049901
TC0400011721.hg_1	MAD2L1	Complex Event	4:120060977–120066662	− 4.485818363	7.26E−06	0.18598516
TC1600009200.hg_3	TRAP1	Complex Event	16:3671791–3674339	− 4.481367645	7.42E−06	0.126216189
TC1100011094.hg_5	UBXN1	Complex Event	11:62678118–62678690	− 4.429188469	9.46E−06	0.11029505
*Top10 down-regulated events*						
TC0600012049.hg_1	GSTA7P	Retained Intron	6:52739711–52741583	5.016013876	5.28E−07	− 0.138058598
TC1600006607.hg_7	ABCA17P	Complex Event	16:2404228–2406828	4.954495463	7.25E−07	− 0.272021512
TC2200008103.hg_1	YPEL1	Retained Intron	22:21701218–21703370	4.882892011	1.05E−06	− 0.249262708
TC0X00008794.hg_1	SLC6A8	Alternative Last Exon	X:153692107–153693041	4.723126799	2.32E−06	− 0.216009533
TC0900008516.hg_4	C9orf91	Retained Intron	9:114637070–114638544	4.644610178	3.41E−06	− 0.201023874
TC0400009396.hg_8	NEIL3	Retained Intron	4:177341642–177351380	4.641704288	3.46E−06	− 0.272342085
TC1700012277.hg_2	CDK5RAP3	Complex Event	17:47974031–47974400	4.504297022	6.66E−06	− 0.209587634
TC1400008749.hg_5	RABGGTA	Retained Intron	14:24266889–24267660	4.491051378	7.09E−06	− 0.216635165
TC0100010297.hg_16	NCSTN	Complex Event	1:160352206–160353160	4.434837994	9.21E−06	− 0.231525085
TC0800009237.hg_15	GPAA1	Alternative 5′ Splice Site	8:144084040–144084134	4.379927673	1.19E−05	− 0.26203504

*PSI* percent-spliced-in

### Differential splicing profiles of RNA-seq datasets

After stringent data processing and filtering, a total of 541 samples (52 normal and 489 tumour samples) and 29,415 AS events were retained in the TGCA-PRAD dataset. Events were categorised into alternate acceptor sites (AA), alternate donor sites (AD), alternate promoter (AP), alternate terminator (AT), exon skip (ES), mutually exclusive exons (ME), and retained intron (RI). Next, a total of 8440 DEAS events from 4257 genes were differentially expressed (Additional file 3: Table S3), including 2372 APs in 1289 genes, 2217 ATs in 1238 genes, 2198 ESs in 1550 genes, 790 RIs in 580 genes, 433 ADs in 376 genes, 394 AAs in 360 genes, and 36 MEs in 35 genes (Additional file 9: Figure S1a and S1b). An upset plot for the DEAS events was generated, indicating certain genes can have up to five AS events and over a third of genes occurred to have exon skipping event (Additional file 9: Figure S1c). Top 30 DEAS events are show as a heatmap in Additional file 9: Figure S1d. We also found that PAK6 had the most significant up-, and down-regulated events and both in AP type (Additional file 9: Figure S1e and S1f, respectively).

The splicing patterns of PCa in PRJEB2449 cohort were analysed using rMATS with a paired statistical model. Our analysis demonstrated that matched tumour and normal samples of 14 PCa patients triggered AS changes in 2131 genes with 3593 significantly regulated events (FDR < 0.05): 497 alternative 3′ splice site (A3SS) events in 375 genes, 185 alternative 5′ splice site (A5SS) events in 168 genes, 283 skipped exon (SE) events in 200 genes, 91 mutually exclusive exons (MXE) events in 67 genes, and 2537 retained intron (RI) events in 1679 genes (Additional file 10: Figure S2a and S2b; Additional file 4: Table S4a–S4f). Moreover, a substantial proportion of the involved parent genes exhibited RI event and some genes had up to five event types (Additional file 10: Figure S2c).

**Fig. 4:**
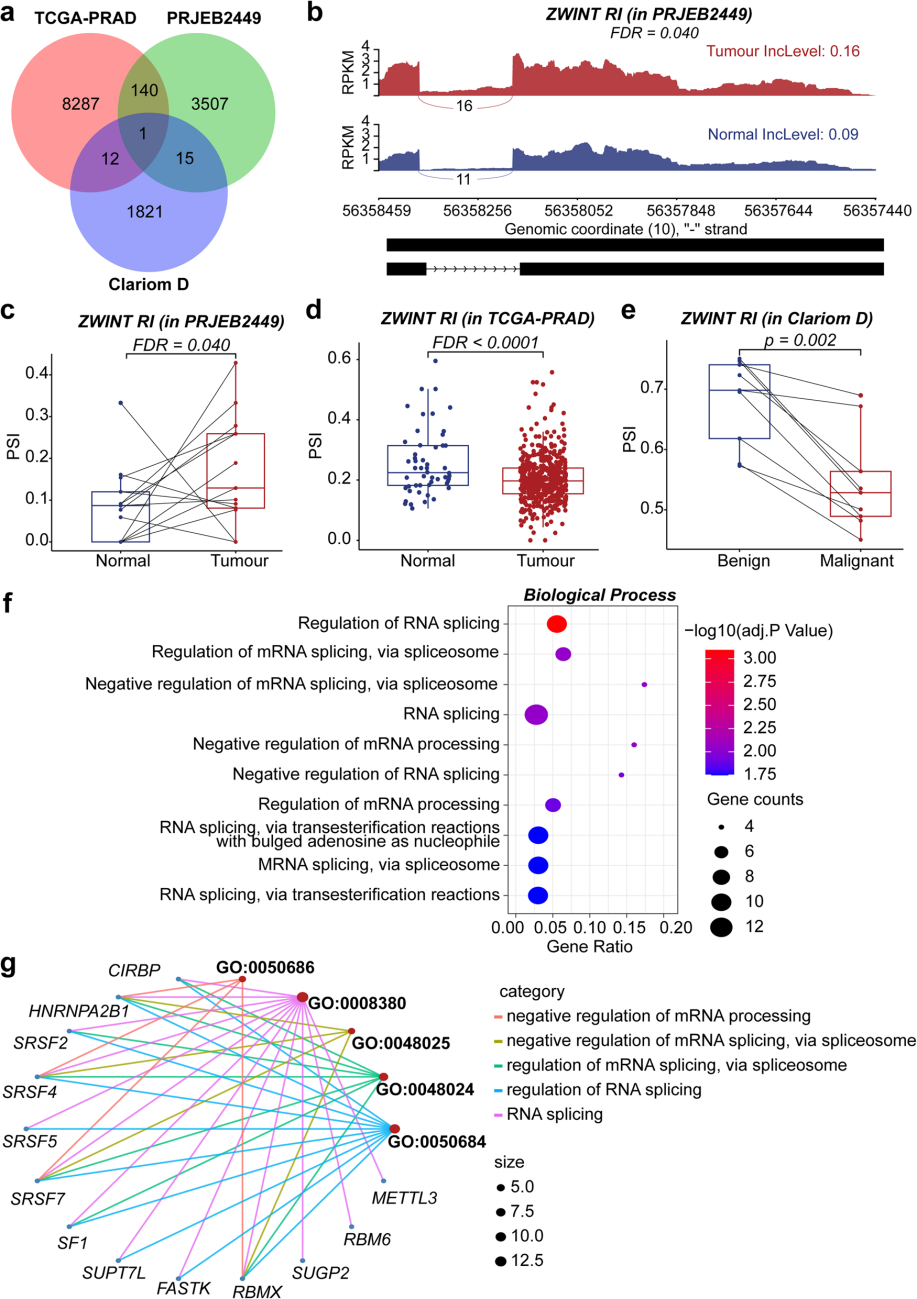
Composite figure demonstrating various analytical aspects of the study. **a** Venn diagram showcasing overlapping differentially expressed alternative splicing (DEAS) events among the three datasets. **b** Sashimi plot of the overlapping *ZWINT* retained intron (RI) event, derived from the PRJEB2449 dataset. The *x*-axis indicates genomics locations, while the *y*-axis indicates normalised fragments per kilobase of transcript per million mapped reads (FPKM) values, averaged across samples within each group. The bulk/ ‘sashimi-like’ region indicates a heavily transcribed, i.e. exonic, region. The gaps between these exonic regions indicate the presence of intronic regions. Red and blue sections symbolise grouped tumour and normal samples, respectively. Junction reads are shown as curved lines crossing the exons, with their numbers indicated on the corresponding curves. The averaged percent-spliced-in (PSI) value, calculated within each sample group, is shown on the right as ‘IncLevel’. The bottom black panel represents the alternative exon–intron structures. **c** Box plot shows the PSI values of the *ZWINT* RI event in the PRJEB2449 dataset, comparing tumour samples with their matched normal counterparts. Boxplots **d** and **e** display the PSI values of the *ZWINT* RI event in the TCGA-PRAD set, comparing tumour samples versus normal ones, and in the Clariom D set, comparing malignant samples versus matched benign ones, respectively. The unadjusted p or the Benjamini–Hochberg (BH) false discovery rate (FDR) values on the box plots were derived from the results of the corresponding differential splicing analyses conducted using the respective tools. **f** Bubble plot of the Gene Ontology Biological Process (GO BP) functional enrichment analysis performed on the parent genes of the 141 DEAS events that overlap between the PRJEB2449 and TCGA-PRAD cohorts. **g** Gene-concept network presents the top five significant terms and the associated genes. *TCGA* The Cancer Genome Atlas, *PRAD* prostate adenocarcinoma

The DEAS events across the Clariom D, TCGA-PRAD, and PRJEB2449 datasets were compared based on their genomic coordinates. Figure 4a summarises the number of common events observed, including overlaps between individual datasets as well as events common to all three datasets. In a comparison between the Clariom D and TCGA-PRAD datasets, we identified common DEAS events: 1 alternative first exon event, 3 cassette exon events, 4 retained intron events, and 5 alternative last exon events (Additional file 5: Table S5a). The Clariom D and PRJEB2449 datasets had 16 overlapping retained intron events (Additional file 5: Table S5b). Notably, only one retained intron event (*ZWINT, chr10: 58117947–58118137, GRCh37*) was found to be significantly expressed across all three datasets (Additional file 5: Table S5c).

Despite this, it showed differential regulation depending on the dataset, with the intron showing a higher inclusion rate in tumour samples relative to normal samples in the PRJEB2449 dataset (Fig. 4b, c) but a decrease in inclusion rate in tumour/malignant samples as compared to normal/benign samples in both the TCGA-PRAD and Clariom D datasets (Fig. 4d, e, respectively). From the two RNA-seq datasets, we identified 141 matching DEAS events (Additional file 5: Table S5d) and further functional analysis revealed these parent genes were significantly enriched in metabolic processes and RNA regulation, including the GO terms ‘regulation of RNA splicing’, ‘regulation of mRNA splicing, via spliceosome’, ‘negative regulation of mRNA splicing, via spliceosome’, and ‘RNA splicing’ (Fig. 4f). Interestingly, splicing factor genes such as *HNRNPA2B1*, *SRSF4*, *SRSF7*, and *RBMX* were found to be highly presented in the top terms (Fig. 4g). The discrepancy in the number of overlapping events across different studies combinations could be attributed to the different profiling technologies and differential splicing tools employed. Furthermore, we elected to exclude the 'complex events', constituting 41% of the total DEAS events, from the Clariom D dataset prior to our overlap analysis. These complex events do not conform easily to the conventional categories of AS events, as they may encompass multiple simultaneous occurrences within the same transcript. Consequently, these complex events complicate the comparison and overlapping of events identified in other datasets.

### Correlation network between splicing factors and BCRFS-associated AS events

**Fig. 5:**
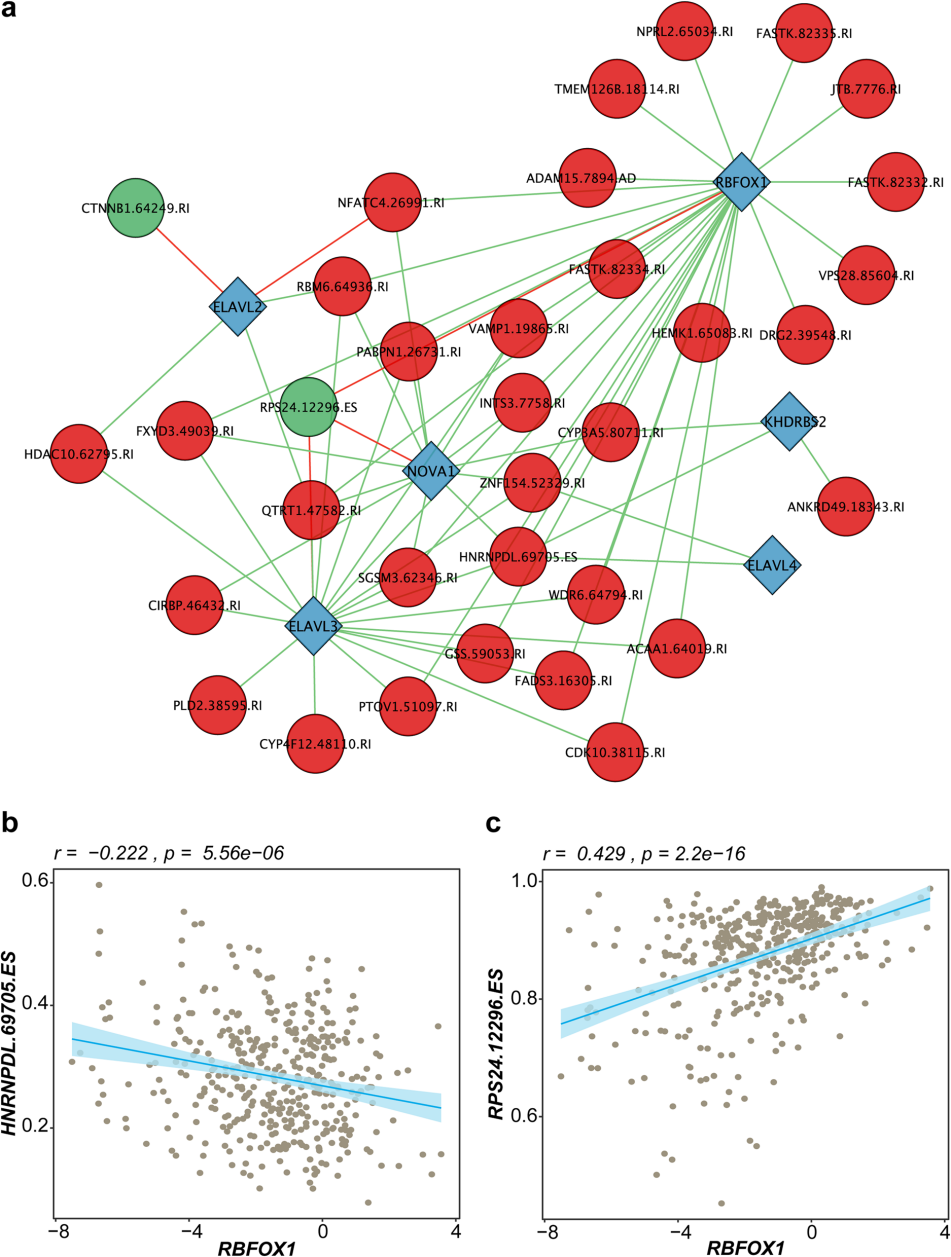
Correlation analysis between differentially expressed splicing factors and prognostic events. **a** Pearson correlation network between differentially expressed splicing factors (DESFs) and biochemical recurrence-free survival (BCRFS)-associated events in the TCGA-PRAD cohort. Blue diamonds denote DESFs, while red and green circles signify events with hazard ratios (HR) greater and less than 1, respectively. Red and green lines denote positive and negative correlations, respectively. **b** A scatter plot visualises the correlation between RBFOX1 and HNRNPDL.69705.ES. **c** A scatter plot represents the correlation between RBFOX1 and RPS24.12296.ES. *TCGA* The Cancer Genome Atlas, *PRAD* prostate adenocarcinoma

In the following work of this study, we considered the 141 overlapping DEAS events across the two RNA-seq datasets as initial event set. In the TCGA-PRAD cohort, 49 splicing factors were found to be differentially expressed between normal and tumour PCa samples via Limma-Voom analysis (Additional file 6: Table S6a), and 41 events were demonstrated significant association with BCRFS (Additional file 6: Table S6b). A correlation network analysis was performed between the differentially expressed splicing factors and the BCRFS-associated events in the TCGA-PRAD dataset (Fig. 5a). This revealed that *RBFOX1*, *ELAVL3* and *NOVA1* were the top three splicing factors correlated with the prognostic events (28, 20 and 13 times, respectively).

Among the prognostic events, *HNRNPDL*.69705.ES emerged as the most interacted risk event (HR > 1), and its PSI value was found to be negatively associated with the expression of all connected SFs. Conversely, *RPS24*.12296.ES was identified as a protective event (HR < 1) that demonstrated a positive correlation with only the top three SFs. Correlation between the most involved splicing factor, *RBFOX1*, and the two top events, HNRNPDL.69705.ES and RPS24.12296.ES, is shown in Fig. 5b, c, respectively.

### Prognostic signature construction and clinical significance

The TCGA-PRAD dataset was divided into training and testing sets for the development of our AS signature. After performing both univariate Cox regression (Additional file 7: Table S7a) and KM survival analysis (Additional file 7: Table S7b), we identified 21 events significantly associated with BCRFS. These events served as candidate events for constructing a prognostic signature model in the TCGA-PRAD training set. Using LASSO (Lambda minimum = 0.01805583; Additional file 11: Figure S3a and S3b) and multivariable COX regression methods, we derived an AS event-based prognostic signature. This consisted of DEAS events in six genes: *CYP4F12, NFATC4, PIGO, CYP3A5, ALS2CL* and *FXYD3*. For each signature event, we generated a sashimi plot using the PRJEB2449 dataset (Additional file 12: Figure S4a–S4f; left panel). The PSI values of signature events showed a significant increase, indicating an up-regulation, in the tumour samples compared to the normal ones in both the PRJEB2449 and TCGA-PRAD datasets (Additional file 12: Figure S4a–S4f; right panel). For each patient, the predictive risk score can be calculated as follows, using the six AS events in the signature along with their corresponding coefficients (Additional file 7: Table S7c):

$$\begin{aligned} {\text{Risk score}} & = \left( {{1}.{37}*{\text{CYP4F12}}|{4811}0|{\text{RI}}} \right) + \left( {{1}.{84}*{\text{NFATC4}}|{26991}|{\text{RI}}} \right) \\ & \quad + \left( { - {4}.{52}*{\text{PIGO}}|{86233}|{\text{RI}}} \right) + \left( {0.{97}0*{\text{CYP3A5}}|{8}0{711}|{\text{RI}}} \right) \\ & \quad + \left( {{1}.0{1}*{\text{ALS2CL}}|{64461}|{\text{RI}}} \right) + ({15}.{6}*{\text{FXYD3}}\left| {{49}0{39}} \right|{\text{RI}}) \\ \end{aligned}$$

Table 3 summarises the sample characteristics in the TCGA-PRAD sets, after removal of records with incomplete clinicopathological information.

**Table 3:** Clinical characteristics of the prostate cancer tumour samples in three TCGA-PRAD sets

Clinical feature	Training set	Validation set	Complete set
	TCGA-PRAD (*n* = 248)	TCGA-PRAD (*n* = 108)	TCGA-PRAD (*n* = 356)
*Age (year) (%)*			
< 60	87 (35.1)	46 (42.6)	133 (37.4)
≥ 60	161 (64.9)	62 (57.4)	223 (62.6)
Biochemical recurrence events (%)	49 (19.8)	15 (13.9)	64 (18.0)
*Pathological T stage (%)*			
T2	85 (34.3)	33 (30.6)	118 (33.1)
T3-4	163 (65.7)	75 (69.4)	238 (66.9)
*Pathological N stage (%)*			
N0	205 (82.7)	85 (78.7)	290 (81.5)
N1	43 (17.3)	23 (21.3)	66 (18.5)
*Gleason score (%)*			
≤ 7	130 (52.4)	59 (54.6)	189 (53.1)
> 7	118 (47.6)	49 (45.4)	167 (46.9)

*TCGA* The Cancer Genome Atlas, *PRAD* prostate adenocarcinoma, *Age* age at diagnosis, *T stage* tumour stage, *N stage* lymph node status (N0 = without lymph node metastasis; N1 = with lymph node metastasis)

Our signature risk score exhibited notable associations with conventional clinicopathological parameters. High risk scores were significantly associated with patients presenting a higher Gleason score (> 7) in the training set (*p* < 0.0001), the testing set (*p* < 0.001) and the complete set (*p* < 0.0001) (Additional file 13: Figure S5b left, middle and right, respectively). Likewise, the signature revealed a significantly elevated risk score in patients with more advanced tumour stages (T3 or T4) across all three TCGA sets (Additional file 13: Figure S5c left (*p* < 0.0001; training set), middle (*p* < 0.05; testing set), and right (*p* < 0.0001; complete set)). Patients under the age of 60 demonstrated significantly lower risk compared to those aged 60 or higher in both the training set (*p* < 0.001) and the complete set (*p* < 0.01) (Additional file 13: Figure S5a left and right, respectively). In both the training and the complete sets, high signature risk scores were significantly associated with patients exhibiting lymph node metastasis (N1) (*p* < 0.001 for both sets; Additional file 13: Figure S5d left and right). Furthermore, univariate Cox regression analysis revealed that our event-based signature had a significant correlation with patients’ BCRFS in the training set (*p* < 0·0001 and Hazard ration (HR) = 2.648 (95% confidence interval (CI): 1.769–3.963); Additional file 14: Figure S6a and Additional file 8: Table S8a), and in the complete set (*p* < 0.0001 and HR = 2.481 (95% CI 1.750–3.516); Additional file 14: Figure S6e and Additional file 8: Table S8c). Multivariate Cox regression indicated that the signature significantly contributed to risk (i.e. it acted as a risk factor with HR > 1) and remained to serve as an independent prognostic factor in both the training set (*p* = 0.003 and HR = 1.931 (95% CI 1.242–3.001); Additional file 14: Figure S6b and Additional file 8: Table S8a), and the complete set (*p* = 0.002 and HR = 1.829 (95% CI 1.256–2.665); Additional file 14: Figure S6f and Additional file 8: Table S8c).

### Prognostic performance of the signature and external validation

**Fig. 6:**
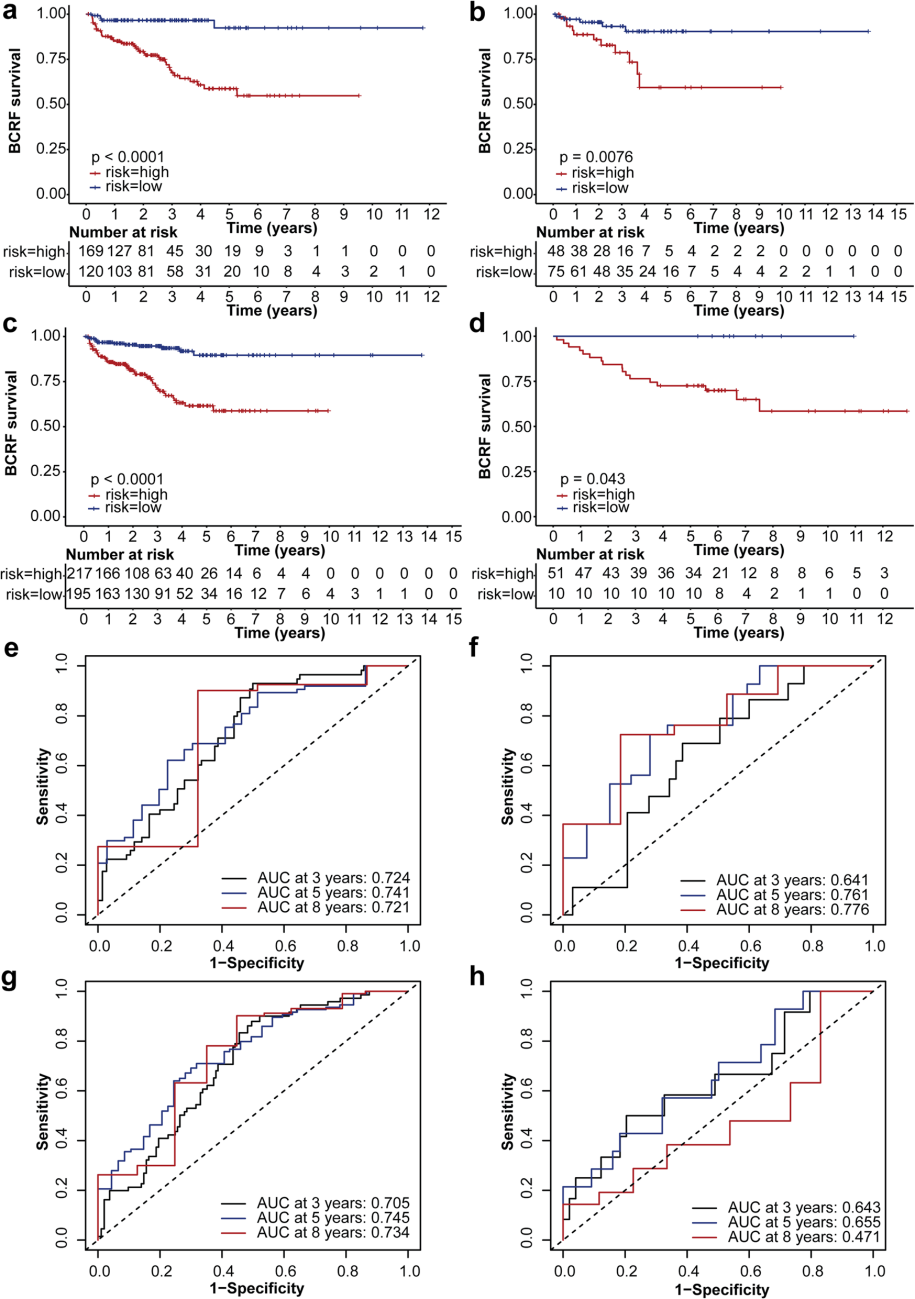
Performance of event-based signature across various datasets. Kaplan–Meier (KM) curves for biochemical recurrence-free survival (BCRFS) in low- and high-risk prostate cancer (PCa) patient groups. Curves are differentiated based on the six prognostic alternative splicing (AS) event signature risk score. These curves are presented for **a** the training set from The Cancer Genome Atlas Prostate Adenocarcinoma (TCGA-PRAD), **b** the testing set, **c** the entire set, and **d** the GSE107299 cohort. Time-dependent receiver operating characteristic (ROC) curves evaluate the performance of the six-event signature for predicting 3-, 5-, and 8-year BCRFS in **e** the TCGA-PRAD training set, **f** the testing set, **g** the complete set, and **h** the GSE107299 cohort

The number of patients experiencing biochemical recurrence increased with the rising event signature risk score, as evidenced by the risk score distribution and biochemical recurrence status of each sample (Additional file 15: Figure S7a–S7c, pertaining to the training set, testing set, and complete set, respectively). The KM survival analysis demonstrated that the signature risk score could significantly differentiate between low- and high-risk groups of patients diagnosed with PCa in the TCGA-PRAD training set (*p* < 0.0001), the testing set (*p* = 0.0076), the complete set (*p* < 0.0001), and the external GSE107299 set (*p* = 0.043) (Fig. 6a–d, respectively). Importantly, these KM survival curves confirmed that patients categorised in the high-risk group exhibited significantly poorer survival compared to patients in the low-risk group. Moreover, the signature demonstrated strong predictive capabilities for 3-, 5-, and 8-year BCRFS in the TCGA-PRAD sets (AUCs: 0.724, 0.741 and 0.721 in the training set; 0.641, 0.761 and 0.776 in the testing set; 0.705, 0.745 and 0.734 in the complete set, respectively; Fig. 6e–g). The corresponding C-index values were 0.701 [95% CI 0.630–0.773], 0.604 [95% CI 0.469–0.739] and 0.679 [95% CI 0.614–0.744]. The prognostic efficacy of the signature was subsequently validated in the external GSE107299 dataset, showcasing relatively good predictive power for both 3- and 5-year BCRFS (AUCs: 0.643 vs. 0.655; Fig. 6h) and a C-index of 0.579 [95% CI 0.449–0.708].

### Nomogram construction and survival performance

**Fig. 7:**
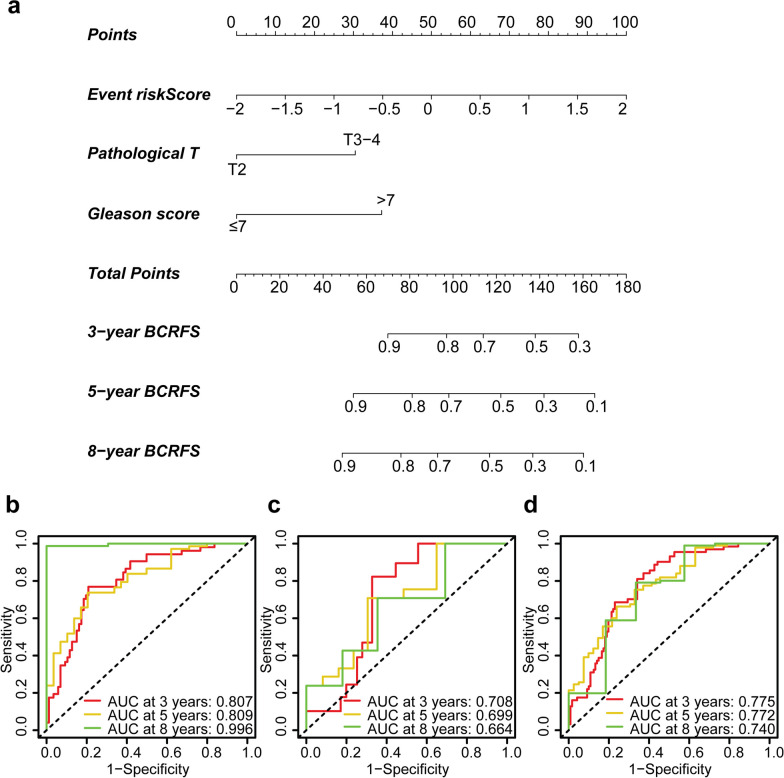
Nomogram and its predictive performance. **a** Nomogram predicting 3-, 5-, and 8-year biochemical recurrence-free survival (BCRFS) for patients diagnosed with prostate cancer (PCa). The performance of the nomogram is evaluated through receiver operating characteristic (ROC) curves in The Cancer Genome Atlas Prostate Adenocarcinoma (TCGA-PRAD) training set (**b**), the testing set (**c**), and the complete set (**d**)

Significant parameters identified through the multivariate Cox regression from the training set were used to construct a nomogram, including the event-based signature risk score, pathological T stage (*p* = 0.044 and HR = 2.497 (95% CI 1.023–6.098)), and Gleason score (*p* = 0.004 and HR = 2.990 (95% CI 1.416–6.312)) (Fig. 7a). The nomogram is a modelling tool that enables individualised predictions. For each patient’s predictor variable, a vertical line is drawn to obtain a corresponding ‘point’. The points from all variables are then summed to generate a ‘total point’, which gives the predicted probability for 3-, 5-, or 8-year BCFRS. The nomogram demonstrated strong predictive power for 3-, 5-, and 8-year BCRFS, as evidenced by the AUCs of 0.807, 0.809 and 0.996 in the training set; 0.708, 0.699 and 0.664 in the testing set; and 0.775, 0.772 and 0.740 in the complete set (Fig. 7b–d, respectively).

**Fig. 8:**
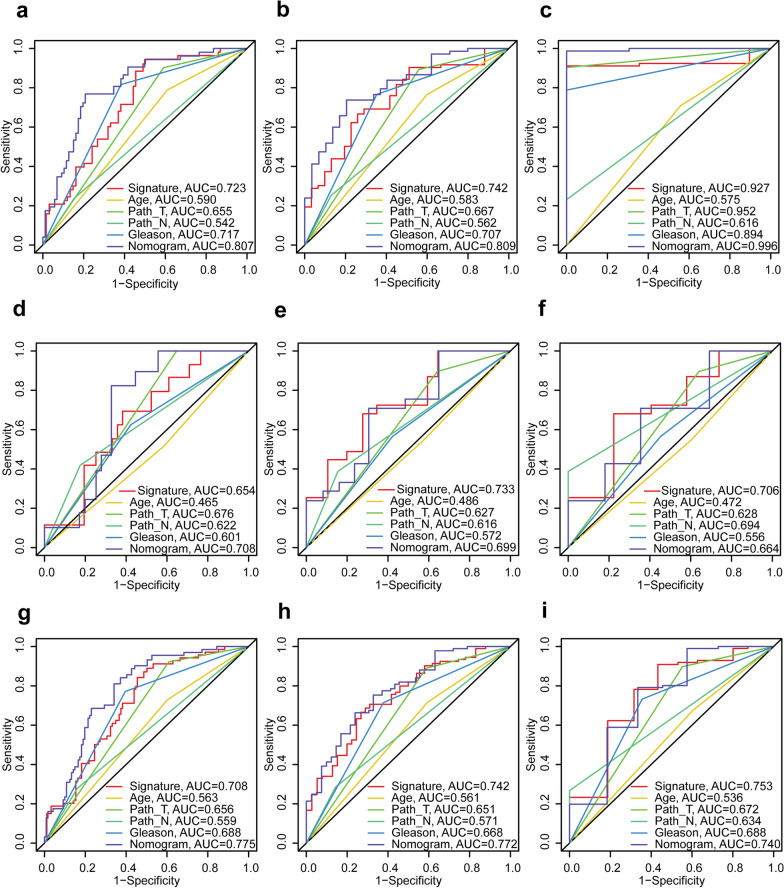
ROC curve comparisons of various model parameters for predicting patient’s 3-, 5-, 8-year BCRFS. Receiver operating characteristic (ROC) curves illustrating 3-, 5-, and 8-year biochemical recurrence-free survival (BCRFS) predictions for patients diagnosed with prostate cancer in The Cancer Genome Atlas Prostate Adenocarcinoma (TCGA-PRAD) training set (**a**, **b**, and **c**, respectively), the testing set (**d**, **e**, and **f**, respectively), and the complete set (**g**, **h**, and **i**, respectively). Different parameters employed are colour-coded and labelled in each subfigure. Age (< 60 vs. ≥ 60): age at diagnosis; Path_T/pathological T stage (T2 vs. T3-T4): tumour stage; Path_N/pathological N stage (N0 vs. N1): lymph node status (N0 = without lymph node metastasis; N1 = with lymph node metastasis); Gleason: Gleason score (≤ 7 vs. > 7)

The corresponding C-index of the nomogram was 0.740 (95% CI 0.670–0.810) in the training set, 0.688 (95% CI 0.604–0.772) in the testing set, and 0.726 (95% CI 0.667–0.784) in the complete set. Our AS event signature and nomogram demonstrated superior predictive performance for 5-year BCRFS compared to all standalone clinicopathological variables across all three TCGA-PRAD sets (Fig. 8b, e, and h). Importantly, these models outperformed both the Gleason score and the patient's age at diagnosis as prognostic markers for BCRFS at all three time points across the three sets (Fig. 8). While the nomogram demonstrated a stronger predictive capability than all other parameters, including the AS event signature, for BCRFS prediction at three distinct time points in the training set (Fig. 8a–c), the AS event signature exceeded the predictive performance of the nomogram for both 5- and 8-year BCRFS in the testing set (Fig. 8e–f) and for 8-year BCRFS in the complete set (Fig. 8i).

## Discussion

Prostate cancer (PCa) is a complex disease with significant clinical challenges due to the heterogeneity of its progression and prognosis. This complexity often limits the effectiveness of existing biomarkers in providing precise prognostic outcomes for individual patients. Acknowledging the important role that AS events play in the tumorigenesis, progression, and advancement of PCa, it is crucial to pinpoint and clarify potential AS events that may serve as valuable prognostic markers. In this study, our overarching aims were threefold; identifying differentially expressed alternative splicing (DEAS) events that overlap across multiple PCa datasets, developing an AS event-based signature model capable of predicting BCRFS, and synthesising potential parameters to establish a nomogram to enhance BCRFS prognosis. Our results revealed several DEAS events across various PCa datasets, with some displaying a significantly association with patients’ BCRFS. A robust signature based on six AS events, alongside a potential clinical nomogram, were developed, exhibiting satisfactory predictive power for patients’ BCRFS. These prognostic models outperformed some traditionally parameters used in PCa, such as Gleason score, for 3-, 5-, and 8-year BCRFS prediction, demonstrating their potential clinical utility.

Dysregulation of AS and its regulatory controls has been revealed to be directly associated with the development, progression, and aggressiveness of PCa [6, 44, 45]. This is well exemplified by dysregulated expression of the *ARv7* isoforms of the androgen receptor (*AR*), which is the is the primary target for early treatment of PCa. *ARv7* exhibits skipping of exon 3 and is constitutively expressed in the nucleus of PCa cells, independent of androgen stimulation [46]. It is the most clinically relevant splicing variants associated with increased biochemical recurrence and poor survival of PCa [47–49]. Another AR variant, *ARv567*, involves the skipping of exons 5–7, is characterised by androgen independence, and exhibits high expression levels in advanced prostate tissue [48, 50]. Other AS events in genes such as *VEGF* [51, 52], *BCL2L1* [53], *SH3GLB1* [54] and *CCDN1* [55–57] have also been demonstrated to play critical roles in PCa development. The afore-mentioned findings substantiate the significance of identifying AS events in genes, which not only contributes to the understanding of potential AS mechanisms, but also enables the discovery of potential diagnostic and/or prognostic biomarkers for PCa, as well as the identification of potential therapeutic targets for treatment.

Our analysis of 141 overlapping DEAS events, obtained from the two RNA-seq datasets both generated using the Illumina HiSeq 2000 platform, led to the identification and subsequent validation of a six-event-based AS signature for BCRFS. The event signature includes intron retention events in the *CYP4F12*, *NFATC4*, *PIGO*, *CYP3A5*, *ALS2CL* and *FXYD3* genes. Our event-based signature exhibits a significant correlation with prevalent clinicopathological factors in PCa, including age at diagnosis, Gleason score, pathological T and N stages, suggesting its potential for clinical applicability. Our AS event signature demonstrated effective performance in predicting 5-year BCRFS. Within the internal training subset of the TCGA-PRAD cohort, the signature model achieved an AUC of 0.741. This performance was consistent in the testing subset (AUC = 0.761) and the complete set (AUC = 0.745) of the same cohort. When applied to the external GSE107299 cohort, our AS event signature also yielded a respectable AUC of 0.655, highlighting its potential generalisability. By utilising Kaplan Meier survival analysis, we found that low-risk patients classified by our signature in both the TCGA-PRAD and GSE107299 sets displayed higher BCRFS rates. Our composite nomogram outperformed all other variables in the TCGA-PRAD training set, while the event signature remained competitive in both testing and complete sets. Furthermore, for predictions at 3-, 5-, and 8-year BCRFS, both our AS event signature and the nomogram displayed superior predictive performance compared to the Gleason score of patients across all three subsets of TCGA-PRAD cohort. Several previous studies have pioneered the development of AS event-based signatures in PCa. For instance, one study [58] developed alternative splicing event signatures based on distinct event types and demonstrated their predictive ability for 5-year disease-free survival (DFS) in PCa patients, using data from the TCGA-PRAD SpliceSeq. These signatures yielded AUCs ranging from 0.380 to 0.761. Upon direct comparison of the retained intron signature identified in the study with our event signature, ours has better performance in predicting 5-year survival (AUC of signature in the study: 0.612 vs. AUC of our signature: 0.741, 0.761, and 0.745 for the three TCGA-PRAD sets, respectively). Another study identified a prognostic signature composed of six AS events of various types, which predicted a 5-year progression-free survival (PFS) in PCa patients with an AUC of 0.793 [15], again using the TCGA-PRAD SpliceSeq data. The slight superiority of this signature over ours could potentially be attributed to the fact that it comprises different types of events. A third study [14] developed a set of AS event type-based signatures from the TCGA-PRAD cohort, with AUCs ranging from 0.663 to 0.868 for predicting DFS. The retained intron based model identified in this third study yielded an AUC of 0.724, which is slightly lower than our 5-year based predictive signature (AUCs: 0.741, 0.761, and 0.745 for the three TCGA-PRAD sets, respectively). However, the majority of existing prognostic AS event signatures in PCa, regardless of the survival endpoint, have been constructed and validated solely from the TCGA-PRAD SpliceSeq dataset, and hence lack either internal or external validation, which calls into question their broad prognostic applicability. Our study aims to bridge this gap by comprehensively investigating AS events across multiple PCa datasets from different platforms, searching for consistency among them at the level of AS events. Furthermore, we developed a prognostic AS event signature, trained and tested both internally and externally, across the selected datasets. To the best of our knowledge, such an approach has not been thoroughly explored in existing literature.

AS events in *PIGO* and *CYP3A5* genes have been relatively understudied. However, *PIGO*, which encodes phosphatidylinositol glycan anchor biosynthesis class O protein, has been reported to be upregulated in prostate tumours, suggesting its role in promoting cell growth [59]. *CYP3A5* encodes the cytochrome p450 3A5 enzyme, involves in xenobiotic metabolism [60]. Genetic variation in *CYP3A5* impacts drug response, which will affect individual response to therapeutic drugs. *CYP3A5* inhibitors can enhance androgen depletion therapy (ADT), while inducers may reduce efficacy [61]; and its polymorphism may specifically decrease the risk of developing low-grade or early stage PCa in the Japanese population [62]. *CYP4F12* encodes cytochrome p450 4F12 and an intron retention event in the *CYP4F12* gene, has been previously demonstrated to hold prognostic value in cervical cancer, being incorporated into a retained intron-based signature model to predict overall survival in patients with cervical cancer [63]. The same retained intron event identified in our study has also been found to be negatively regulated in both left- and right-sided colon tumour tissues compared to normal tissues [64], suggesting a wider impact of *CYP4F12* dysregulation on cancer pathogenesis. *NFATC4* encodes a protein called nuclear factor of activated T cells, a transcription factor involves in immune response, cell growth, and differentiation. The same intron retention event in the *NFATC4* gene we identified in our study has been implicated in prognosis in other cancer types. In papillary thyroid cancer, it predicted patients’ progression-free survival (PFS) [65], and in glioblastoma, it was considered as a key event in predicting overall survival [66]. Whilst the alternate donor site (AD) event in *NFATC4* has been identified as a poor prognostic indicator for overall survival in gastric cancer [67], its association with overall survival appears to be the opposite in pancreatic cancer [68]. These findings highlight the complex and context-dependent nature of splicing events in cancer, as the same event can have different prognostic implications in different types of cancer. splicing alterations could impact cancer pathogenesis by regulating essential pathways. *ALS2CL* encodes a guanine-nucleotide exchange factor for Rab5 and acts as a modulator for endosome dynamics [69]. Its role in cancer is relatively unexplored, but research links alternative splicing at this locus to colorectal cancer [70, 71]. Alternative splicing events in *ALS2CL* are upregulated in primary tumours compared to normal tissues and may be prognostic markers for overall survival and disease-free survival (DFS) [70, 71]. *FXYD3*, encodes the FXYD domain-containing ion transport regulator 3A and regulates ion transport activity. The alternate promoter (AP) event identified in *FXYD3* has been reported to be a significant overall survival predictor in lung cancer [72]. It has been demonstrated to be over-expressed in prostate tumour samples and it is important for proliferation in prostate carcinomas [73, 74].

An interesting observation in our data is the abundance of dysregulated splicing events involving retained introns; of the 141 DEAS events shared between datasets, 115 were retained intron events. Retained introns may be unreliably detected in short read NGS data [75] and as such are likely to have been underrepresented in such datasets. RI events can arise from mutation, epigenetic change or splicing factor dysregulation, resulting in a failure to properly recognise splice sites. The consequences of this may be the production of aberrant out-of-frame transcripts, which are subject to nonsense-mediated decay. If not degraded at the transcript level by NMD, proteins may be produced with inappropriate amino acid inserts, or changes to the amino acid sequence after the splicing error that bear no resemblance to the consensus sequence for the gene. As such, one would expect them to be deleterious for cell, tissue and organ function, and accordingly, they have been demonstrated to be a feature of cancer development and therapeutic resistance in cancer [76], and in particular, a hallmark of stemness associated with aggressiveness in prostate cancer itself [5]. One prominent example of this is our data is an intron inclusion event in the *ZWINT* gene, that is common to all of the datasets we examined. *ZWINT* appears as a hub gene in our PPI network and encodes a fundamental component of the mitotic checkpoint and has been previously implicated in overall and disease-free survival of lung cancer [77]. *ZWINT* encodes the ZW10 interacting protein, a known AR target gene [78] and component of the kinetochore at the mitotic spindle checkpoint which has previously been reported as an independent prognostic marker for PCa [77]. Furthermore, silencing *ZWINT* expression leads to downregulation of positive cell cycle regulators such as *CCND1*, *CCNE1* and *CDK4* [79]. Introns in the 3′ UTR are frequently associated with the poison exons, which cause degradation of the transcripts containing them, and are a potent component of endogenous gene regulation [80]. Thus, the exclusion *ZWINT* intron in the tumour samples is predicted to lead to higher levels of total *ZWINT* expression, consequent elevation of cyclin D1 and E1 and cyclin dependent kinase 4 expression and promotion of cell proliferation.

Splice site usage is controlled by the combinational binding of splicing factor proteins to exon and intron splicing enhancer and silencer motifs around the splice sites [81]. A splicing factor can therefore regulate alternative splicing of thousands of genes. Analysing coordinate changes in splicing factor expression in correlation with alternative splicing events may provide insights into the mechanistic basis of individual isoform usage. From our correlation network analysis between differentially expressed splicing factors and survival-associated alternative splicing events, transcripts encoding *RBFOX1*, *ELAVL3*, and *NOVA1* emerged as the top three splicing factors most correlated with survival associated alternative splicing changes. *RBFOX1* (RNA binding fox-1 homolog 1) encodes an RNA-binding protein involved in post-transcriptional regulation, including AS of genes related to cell functions such as proliferation and apoptosis. It suppresses malignancy in glioma by regulating *TPM1* splicing [82], linked to various cancer types, including breast cancer [83], lung cancer [84], and prostate cancer [85]. Additionally, *RBFOX1*, like many splicing factors, also has roles in the stabilisation of mRNA by binding to 3′UTR regions, and its loss correlates with poor glioblastoma (GBM) patient survival [86]. *NOVA1* (NOVA alternative splicing regulator 1), a well-known regulator of alternative splicing first identified in lung cancer cells [87], modulates pre-mRNA splicing in genes related to neuronal function and cancer progression [88, 89]. Dysregulated *NOVA1*-mediated splicing is linked to various cancers, including colorectal [90], pancreatic [91], lung [92], and prostate [93]. *NOVA1* has previously been described to be significantly up-regulated in PC-3 PCa cell lines) and in both in vitro and in vivo models, at the levels of mRNA and protein [94]. Over-expression of *NOVA1* has also been identified as a key SF directly associated with the aggressiveness of PCa [95]. *ELAVL3* (ELAV like RNA binding protein 3), an RNA-binding protein regulating post-transcriptional gene expression, is mainly expressed in neuronal cells [96] and found as a potential mRNA marker in small cell lung cancer patients [97]. Its precise role in prostate cancer (PCa) development and progression remains uncertain, necessitating further research to clarify its involvement in the disease.

Through our correlation network analysis, we identified exon skipping events in *HNRNPDL* and *RPS24* as the two most prominent events regulating the splicing of other splicing factors. Exon skipping in the *HNRNPDL* gene emerged as a risk event (HR > 1) and exhibited a negative correlation with all connected splicing factors, whereas a skipping event in the ribosomal protein subunit *RPS24* was the most interacted protective factor (HR < 1) and positively associated with the top three interacting SFs in the network. *HNRNPDL* (heterogeneous nuclear ribonucleoprotein D-like protein), has previously been implicated it in tumour development and progression, with studies highlighting its role in abnormal cell proliferation in PCa cells [98] and regulation of transcription and alternative splicing of genes related to tumorigenesis, including cell death, proliferation, migration, and the JAK-STAT pathway [99]. *RPS24* (ribosomal protein S24) encodes a ribosomal protein crucial for ribosome formation. As a potential malignancy biomarker, *RPS24* is over-expressed in malignant PCa tissues [100]. Notably, in line with the event identified in our study, exon 5 of *RPS24*, an *ESRP2*-repressed exon, is frequently skipped in prostate tumour tissue [101] and correlates with hypoxia in PCa samples [102], suggesting its AS may serve as a tumour hypoxia marker.

## Conclusions

In conclusion, we have defined alternative splicing events that are shared between multiple prostate cancer datasets and carried out a systematic and thorough analysis of these in relation to BCRFS. We have developed a unique six-event-based signature and a nomogram, incorporating the event signature, pathological T stage, and Gleason score, which demonstrated satisfactory predictive ability for BCRFS in PCa patients which was superior to the predictive capabilities of commonly employed clinicopathological factors at the 5-year time point. More research is necessary to validate the clinical significance of the observed AS events and understand their underlying molecular mechanisms. This knowledge may facilitate the identification of potential prognostic AS event candidates and the development of more precise and personalised therapeutic targets for PCa.

### Supplementary Information

**Additional file 1: Table S1**. Differential alternative splicing events from EventPointer analysis in the Clariom D dataset.

**Additional file 2:Table S2**. CancerMine results.

**Additional file 3: Table S3**. Differentially expressed alternative splicing events identified in the TCGA-PRAD dataset. TCGA: The Cancer Genome Atlas; PRAD: prostate adenocarcinoma; ID: event identifier; logFC: log2 transformed fold change; AveExpr: average expression (log2-transformed) for the event over all samples; t: moderated t-statistic; P.Value: raw p-value; adj.P.Val:adjusted p-value using Benjamini-Hochberg (BH) approach; B: log-odds; gene: parent gene symbol of the event; asType: event type; ID2: alternative identifier; AA: alternate acceptor sites; AD: alternate donor sites; AP: alternate promoter; AT: alternate terminator; ES: exon skip; ME: mutually exclusive exons; RI: retained intron.

**Additional file 4: Table S4**. rMATS results for PRJEB2449 cohort. (a) A summary of the total number of events, significant annotated splicing events, and their associated parent genes as identified using rMATS in the PRJEB2449 dataset. rMATS: Replicate Multivariate Analysis of Transcript Splicing. **(b)** rMATS output for significant alternative 3′ splice site (A3SS) events. rMATS: Replicate Multivariate Analysis of Transcript Splicing. **(c)** rMATS output for significant alternative 5′ splice site (A5SS) events. rMATS: Replicate Multivariate Analysis of Transcript Splicing. **(d)** rMATS output for significant mutually exclusive exons (MXE) events. rMATS: Replicate Multivariate Analysis of Transcript Splicing. **(e)** rMATS output for significant retained intron (RI) events. rMATS: Replicate Multivariate Analysis of Transcript Splicing. **(f)** rMATS output for significant skipped exon (SE) events. rMATS: Replicate Multivariate Analysis of Transcript Splicing.

**Additional file 5: Table S5**. Overlapping events among the cohorts.(a) Overlapping differentially expressed alternative spicing events between the Clariom D and TCGA-PRAD datasets. TCGA: The Cancer Genome Atlas; PRAD: prostate adenocarcinoma. **(b)** Overlapping differentially expressed alternative spicing events between the Clariom D and PRJEB2449 datasets. **(c)** Overlapping differentially expressed alternative spicing events across the Clariom D, TCGA-PRAD and PRJEB2449 datasets. TCGA: The Cancer Genome Atlas; PRAD: prostate adenocarcinoma. **(d)** Overlapping differentially expressed alternative spicing events observed between the TCGA-PRAD and PRJEB2449 datasets. TCGA: The Cancer Genome Atlas; PRAD: prostate adenocarcinoma.

**Additional file 6: Table S6**. Differentially expressed splicing factors and prognostic alternative splicing events. (a) Results of the differential gene expression analysis of the splicing factors in the TCCGA-PRAD dataset. TCGA: The Cancer Genome Atlas; PRAD: prostate adenocarcinoma; logFC: log2 transformed fold change; AveExpr: average expression (log2-transformed) for the gene over all samples; t: moderated t-statistic; P.Value: raw p-value; adj.P.Val:adjusted p-value using Benjamini-Hochberg (BH) approach; B: log-odds. **(b)** Results of the univariate Cox regression analysis of the biochemical recurrence-free survival (BCRFS)-associated events in the TCGA-PRAD datasets. TCGA: The Cancer Genome Atlas; PRAD: prostate adenocarcinoma; z: the values of the Wald statistic; HR: hazard ratio of the event; HR.95L: the lower limit of 95% confidence interval; HR.95H: the upper limit of the 95% confidence interval; p: p-values obtained from the likelihood ratio test.

**Additional file 7: Table S7**. Screening for candidate prognostic events associated with BCRFS. (a) Univariate Cox regression analysis of overlapping differentially expressed alternative splicing (DEAS) events identified in both The Cancer Genome Atlas-Prostate Adenocarcinoma (TCGA-PRAD) and PRJEB2449 cohorts, as related to the biochemical recurrence-free survival (BCRFS) of prostate cancer (PCa) patients.z: the values of the Wald statistic; HR: hazard ratio of the event; HR.95L: the lower limit of 95% confidence interval; HR.95H: the upper limit of the 95% confidence interval; p: p-values obtained from the likelihood ratio test. **(b)** Kaplan–Meier analysis of the overlapping differentially expressed alternative splicing (DEAS) events identified in both The Cancer Genome Atlas-Prostate Adenocarcinoma (TCGA-PRAD) and PRJEB2449 cohorts, with regard to the biochemical recurrence-free survival (BCRFS) of prostate cancer (PCa) patients. 'p-value' refers to the statistical significance as determined by the log-rank test. **(c)** Multivariate Cox regression analysis of the 21 intersected events identified from both the univariate Cox regression and the Kaplan–Meier (KM) analyses, pertaining to the biochemical recurrence-free survival (BCRFS) of prostate cancer (PCa) patients. The coefficients are represented by 'coef'; HR: hazard ratio of the event; HR.95L: the lower limit of 95% confidence interval; HR.95H: the upper limit of the 95% confidence interval; p: p-values obtained from the likelihood ratio test.

**Additional file 8: Table S8**. Cox regression between our event signature and traditional clinicopathological parameters. (a) Univariate and multivariate Cox regression of the alternative splicing event signature and clinicopathological factors with respect to the biochemical recurrence-free survival of prostate cancer patients in the TCGA-PRAD training set. TCGA: The Cancer Genome Atlas; PRAD: prostate adenocarcinoma; Age: age at diagnosis; T stage: tumour stage; N stage: lymph node status (N0 = without lymph node metastasis; N1 = with lymph node metastasis). HR: hazard ratio of the parameter; HR.95L: the lower limit of 95% confidence interval; HR.95H: the upper limit of the 95% confidence interval; p: p-values obtained from the likelihood ratio test. **(b)** Univariate and multivariate Cox regression of the alternative splicing event signature and clinicopathological factors with respect to the biochemical recurrence-free survival of prostate cancer patients in the TCGA-PRAD testing set. TCGA: The Cancer Genome Atlas; PRAD: prostate adenocarcinoma; Age: age at diagnosis; T stage: tumour stage; N stage: lymph node status (N0 = without lymph node metastasis; N1 = with lymph node metastasis). HR: hazard ratio of the parameter; HR.95L: the lower limit of 95% confidence interval; HR.95H: the upper limit of the 95% confidence interval; p: p-values obtained from the likelihood ratio test. **(c)** Univariate and multivariate Cox regression of the alternative splicing event signature and clinicopathological factors with respect to the biochemical recurrence-free survival of prostate cancer patients in the TCGA-PRAD complete set. TCGA: The Cancer Genome Atlas; PRAD: prostate adenocarcinoma; Age: age at diagnosis; T stage: tumour stage; N stage: lymph node status (N0 = without lymph node metastasis; N1 = with lymph node metastasis). HR: hazard ratio of the parameter; HR.95L: the lower limit of 95% confidence interval; HR.95H: the upper limit of the 95% confidence interval; p: p-values obtained from the likelihood ratio test..

**Additional file 9: Figure S1**. Overview of DEAS events identified between tumour and normal prostate samples within the TCGA-PRAD cohort. (a) Circle plot illustrates the count and proportion of the differentially expressed alternative splicing (DEAS) events across each event type. **(b)** Circle plot represents the number of parent genes involved in each event type among the DEAS events. **(c)** UpSet plot elucidates the DEAS event parent genes, indicating the number of genes engaged in distinct event types (illustrated by horizontal bars) and their involvement in various event type combinations (represented by vertical bars and connected red dots). **(d)** Heatmap presents the percent-spliced-in (PSI) values of the top 30 DEAS events, scaled and clustered by rows (i.e. events). The heatmap’s colour intensity, transitioning from blue (least expressed) to red (highest expressed), signifies scaled PSI values. **(e)** Box plot of the most significant up-regulated event. **(f)** Box plot of the most significant down-regulated event. TCGA: The Cancer Genome Atlas; PRAD: prostate adenocarcinoma; AA: alternate acceptor sites; AD: alternate donor sites; AP: alternate promoter; AT: alternate terminator; ES: exon skip; ME: mutually exclusive exons; RI: retained intron.

**Additional file 10: Figure S2**. Overview of DEAS events identified between tumour and matched normal prostate samples: PRJEB2449 cohort. (a) Circle plot illustrates the count and proportion of the differentially expressed alternative splicing (DEAS) events across each event type. **(b)** Circle plot represents the number of parent genes involved in each event type among the DEAS events. **(c)** UpSet plot elucidates the DEAS event parent genes, indicating the number of genes engaged in distinct event types (illustrated by horizontal bars) and their involvement in various event type combinations (represented by vertical bars and connected red dots). A3SS: Alternative 3′ splice site; A5SS: Alternative 5′ splice site; SE: Skipped exon; MXE: mutually exclusive exons; RI: retained intron.

**Additional file 11: Figure S3**. LASSO regression for the selection and identification of biochemical recurrence-free survival (BCRFS)-associated events. (a) Determination of optimal Lambda values. **(b)** Coefficient profiles for all evaluated genes. LASSO: least absolute shrinkage and selection operator.

**Additional file 12: Figure S4**. Overview of the six events from the prognostic signature. Sashimi and box plots of the six retained intron (RI) events in **(a)** *CYP4F12*, **(b)** *NFATC4*, **(c)** *PIGO*, **(d)** *CYP3A5*, **(e)** *ALS2CL*, and **(f)** *FXYD3*. Sashimi plots (left panel) were derived from the PRJEB2449 dataset, with their explanations as in Fig. 4. The box plots demonstrate the differences in percent-spliced-in (PSI) values between normal and tumour prostate samples in the TCGA-PRAD set (right upper panel), and between matched normal and tumour samples in the PRJEB2449 set (right lower panel). The Benjamini-Hochberg (BH) false discovery rate (FDR) values on the box plots were derived from the results of the corresponding differential splicing analyses conducted using the respective tools. TCGA: The Cancer Genome Atlas; PRAD: prostate adenocarcinoma.

**Additional file 13: Figure S5**. Clinical impact of the splicing event signature. Box plots of the alternative splicing event-based risk score in relation to **(a)** patient age at diagnosis (< 60 vs. ≥ 60), **(b)** Gleason score (≤ 7 vs. > 7), **(c)** pathological T stage (T2 vs. T3-T4), and **(d)** pathological N stage (N0 vs. N1). These plots are provided for the TCGA-PRAD training set (left panel), the testing set (middle panel) and the complete set (right panel). The significance of the risk score difference between the two groups is denoted with asterisks (ns, no significance, * p < 0.05, ** p < 0.01, *** p < 0.001, **** p < 0.0001). Pathological T stage: tumour stage; Pathological N stage: lymph node status (N0 = without lymph node metastasis; N1 = with lymph node metastasis).

**Additional file 14: Figure S6**. Assessment of the potential independent prognostic factors. Univariate and multivariate Cox regression analyses of alternative splicing event-based signature risk score and various clinicopathological variables, including age of the patient at diagnosis with prostate cancer (< 60 vs. ≥ 60), pathological T stage (tumour stage; T2 vs. T3-T4), pathological N stage (Lymph node status; N0 vs. N1) and Gleason score (≤ 7 vs. > 7). Forest plots of the respective univariate Cox and multivariate Cox in the TCGA-PRAD training set **(a, b)**, the testing set **(c, d)**, and the complete set **(e, f)**. TCGA: The Cancer Genome Atlas; PRAD: prostate adenocarcinoma.

**Additional file 15: Figure S7**. Risk score distribution (upper) and biochemical recurrence classification (recurred or not) in PCa patients (lower).These classifications are presented for the TCGA-PRAD training set **(a)**, the testing set **(b)**, and the complete set **(c)**. PCa: prostate cancer; TCGA: The Cancer Genome Atlas; PRAD: prostate adenocarcinoma.

## Data Availability

The gene expression profiling procedure using the Affymetrix Clariom D Human Array for this study is detailed in our previous paper [22]. The corresponding raw CEL data have been submitted to the Gene Expression Omnibus (GEO) database, under accession number GSE246282. Percent-spliced-in (PSI) values of alternative splicing events for TCGA-PRAD cohort were available at TCGA SpliceSeq (https://bioinformatics.mdanderson.org/TCGASpliceSeq/index.jsp). Raw RNA-seq data for PRJEB2449 cohort were available at ENA (https://www.ebi.ac.uk/ena/browser/home). Raw CEL files of microarray data for GSE107299 cohort were available at GEO (https://www.ncbi.nlm.nih.gov/geo/).
