# Supplementary figures and images for: Comprehensive analysis of alternative splicing across multiple transcriptomic cohorts reveals prognostic signatures in prostate cancer
Source: Hum Genomics. 2023 Nov 3;17:97. doi: 10.1186/s40246-023-00545-w (PMC10623736; doi:10.1186/s40246-023-00545-w)

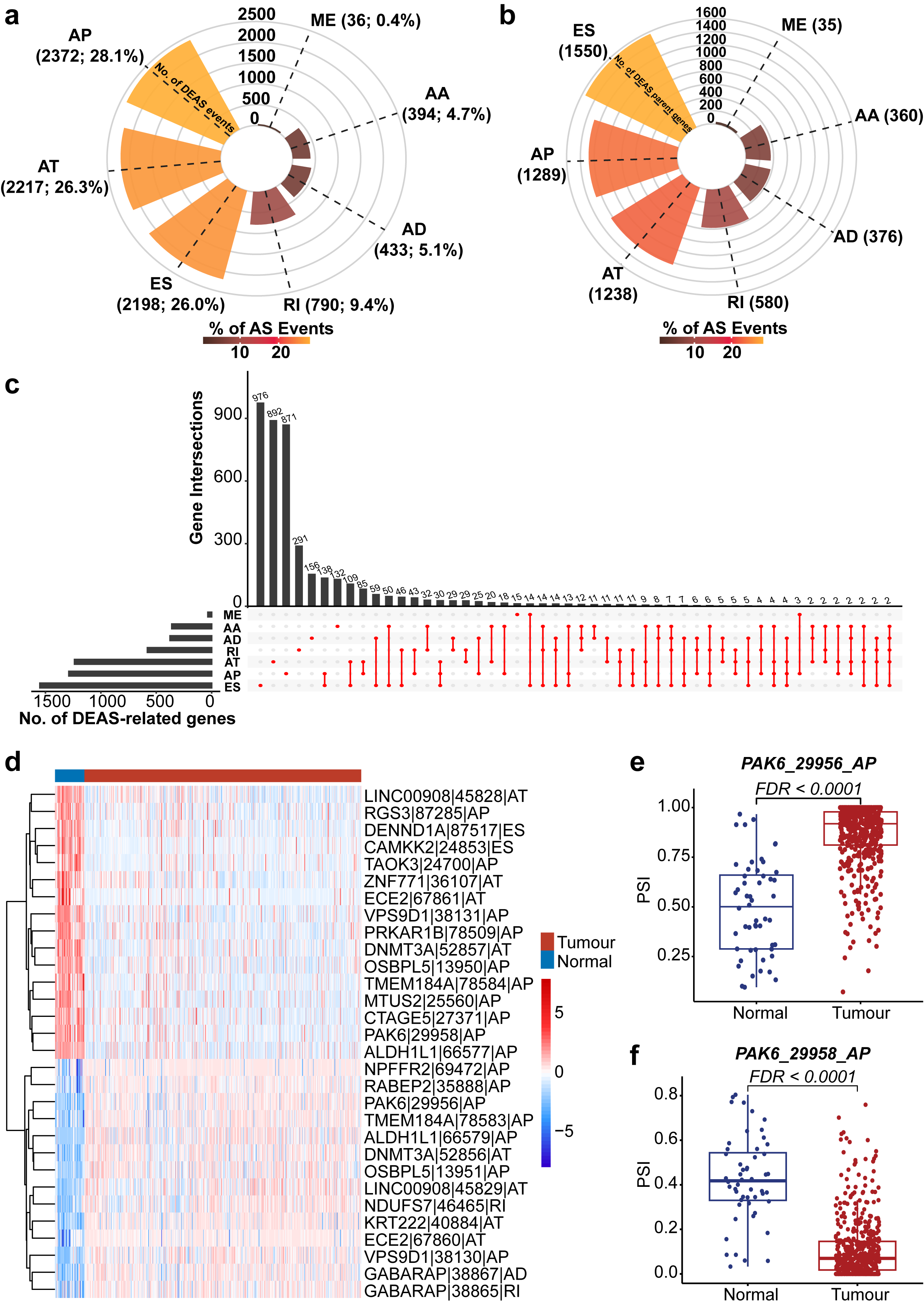

Supplement: Supplementary file 9 — Additional file 9: Figure S1. Overview of DEAS events identified between tumour and normal prostate samples within the TCGA-PRAD cohort. (a) Circle plot illustrates the count and proportion of the differentially expressed alternative splicing (DEAS) events across each event type. (b) Circle plot represents the number of parent genes involved in each event type among the DEAS events. (c) UpSet plot elucidates the DEAS event parent genes, indicating the number of genes engaged in distinct event types (illustrated by horizontal bars) and their involvement in various event type combinations (represented by vertical bars and connected red dots). (d) Heatmap presents the percent-spliced-in (PSI) values of the top 30 DEAS events, scaled and clustered by rows (i.e. events). The heatmap’s colour intensity, transitioning from blue (least expressed) to red (highest expressed), signifies scaled PSI values. (e) Box plot of the most significant up-regulated event. (f) Box plot of the most significant down-regulated event. TCGA: The Cancer Genome Atlas; PRAD: prostate adenocarcinoma; AA: alternate acceptor sites; AD: alternate donor sites; AP: alternate promoter; AT: alternate terminator; ES: exon skip; ME: mutually exclusive exons; RI: retained intron. [file 40246_2023_545_MOESM9_ESM.tif]

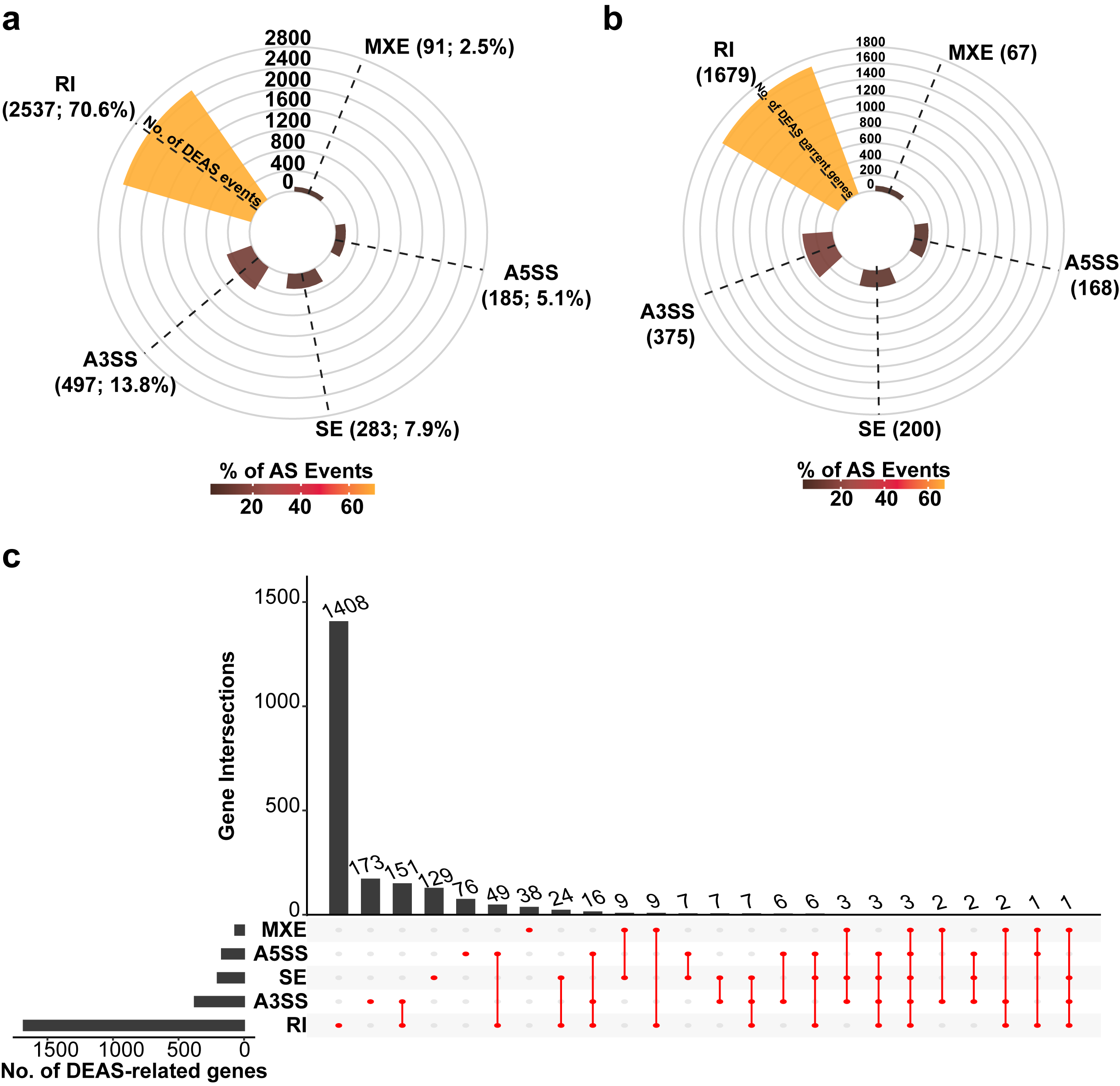

Supplement: Supplementary file 10 — Additional file 10: Figure S2. Overview of DEAS events identified between tumour and matched normal prostate samples: PRJEB2449 cohort. (a) Circle plot illustrates the count and proportion of the differentially expressed alternative splicing (DEAS) events across each event type. (b) Circle plot represents the number of parent genes involved in each event type among the DEAS events. (c) UpSet plot elucidates the DEAS event parent genes, indicating the number of genes engaged in distinct event types (illustrated by horizontal bars) and their involvement in various event type combinations (represented by vertical bars and connected red dots). A3SS: Alternative 3′ splice site; A5SS: Alternative 5′ splice site; SE: Skipped exon; MXE: mutually exclusive exons; RI: retained intron. [file 40246_2023_545_MOESM10_ESM.tif]

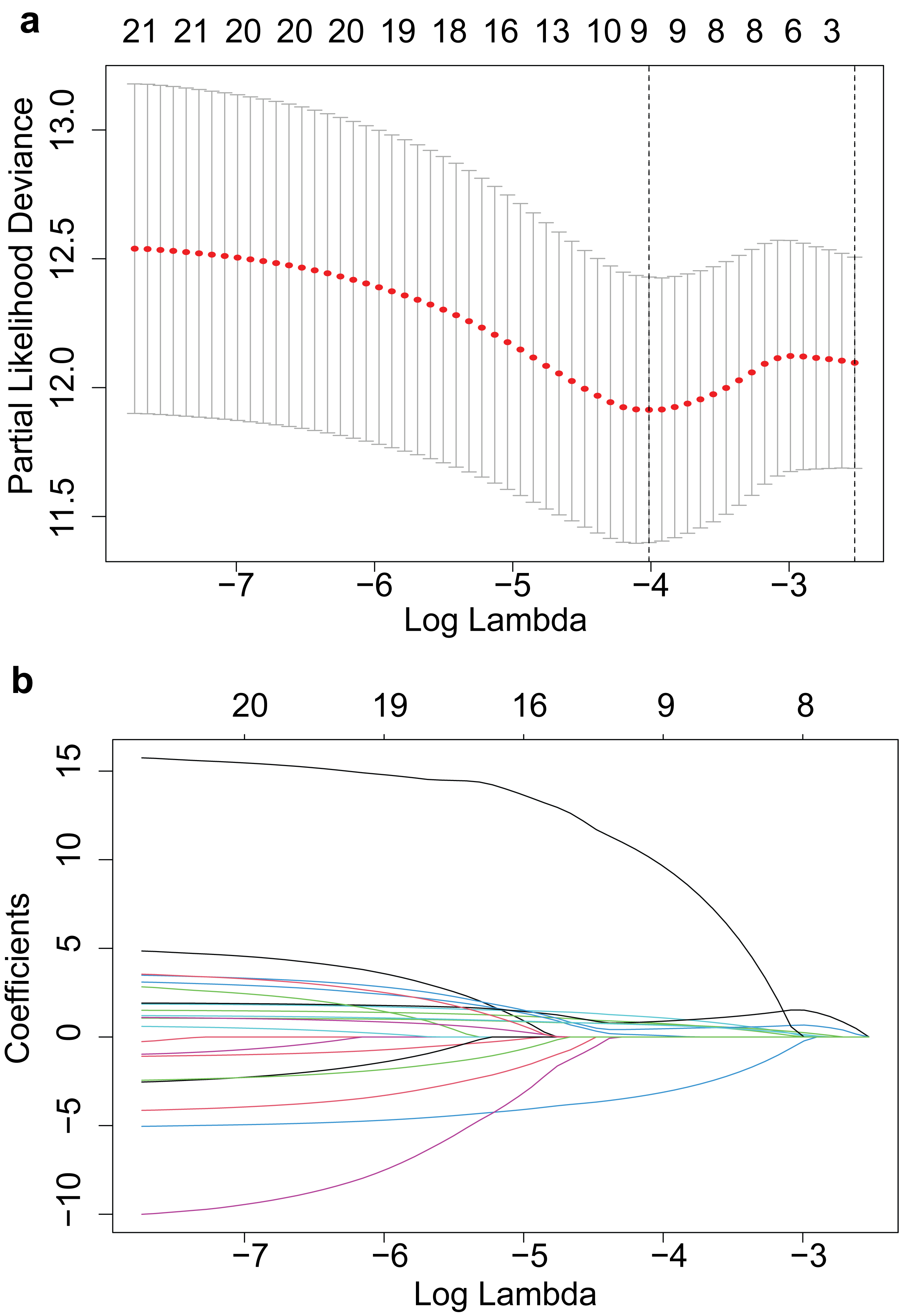

Supplement: Supplementary file 11 — Additional file 11: Figure S3. LASSO regression for the selection and identification of biochemical recurrence-free survival (BCRFS)-associated events. (a) Determination of optimal Lambda values. (b) Coefficient profiles for all evaluated genes. LASSO: least absolute shrinkage and selection operator. [file 40246_2023_545_MOESM11_ESM.tif]

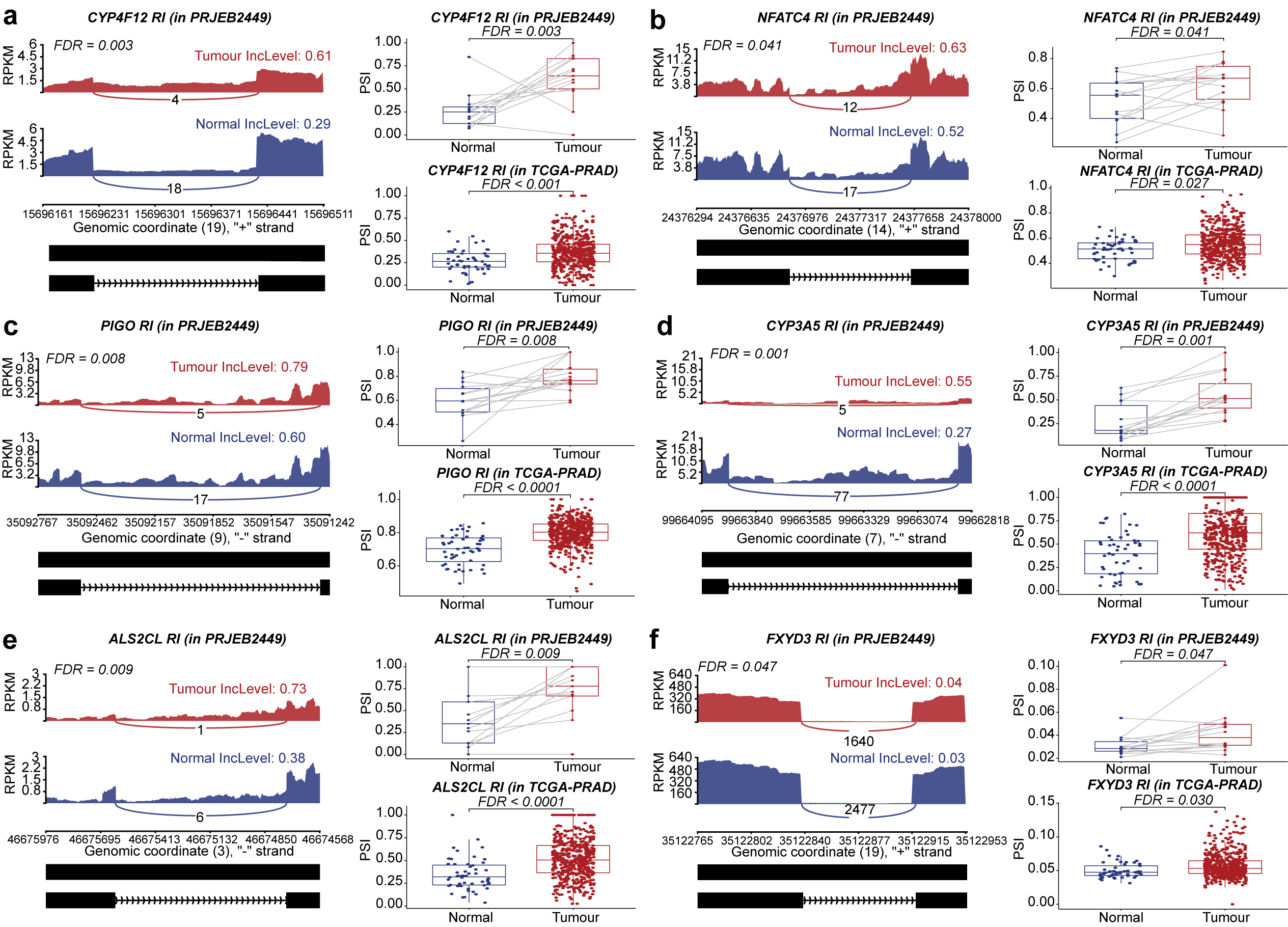

Supplement: Supplementary file 12 — Additional file 12: Figure S4. Overview of the six events from the prognostic signature. Sashimi and box plots of the six retained intron (RI) events in (a) CYP4F12, (b) NFATC4, (c) PIGO, (d) CYP3A5, (e) ALS2CL, and (f) FXYD3. Sashimi plots (left panel) were derived from the PRJEB2449 dataset, with their explanations as in Fig. 4. The box plots demonstrate the differences in percent-spliced-in (PSI) values between normal and tumour prostate samples in the TCGA-PRAD set (right upper panel), and between matched normal and tumour samples in the PRJEB2449 set (right lower panel). The Benjamini-Hochberg (BH) false discovery rate (FDR) values on the box plots were derived from the results of the corresponding differential splicing analyses conducted using the respective tools. TCGA: The Cancer Genome Atlas; PRAD: prostate adenocarcinoma. [file 40246_2023_545_MOESM12_ESM.tif]

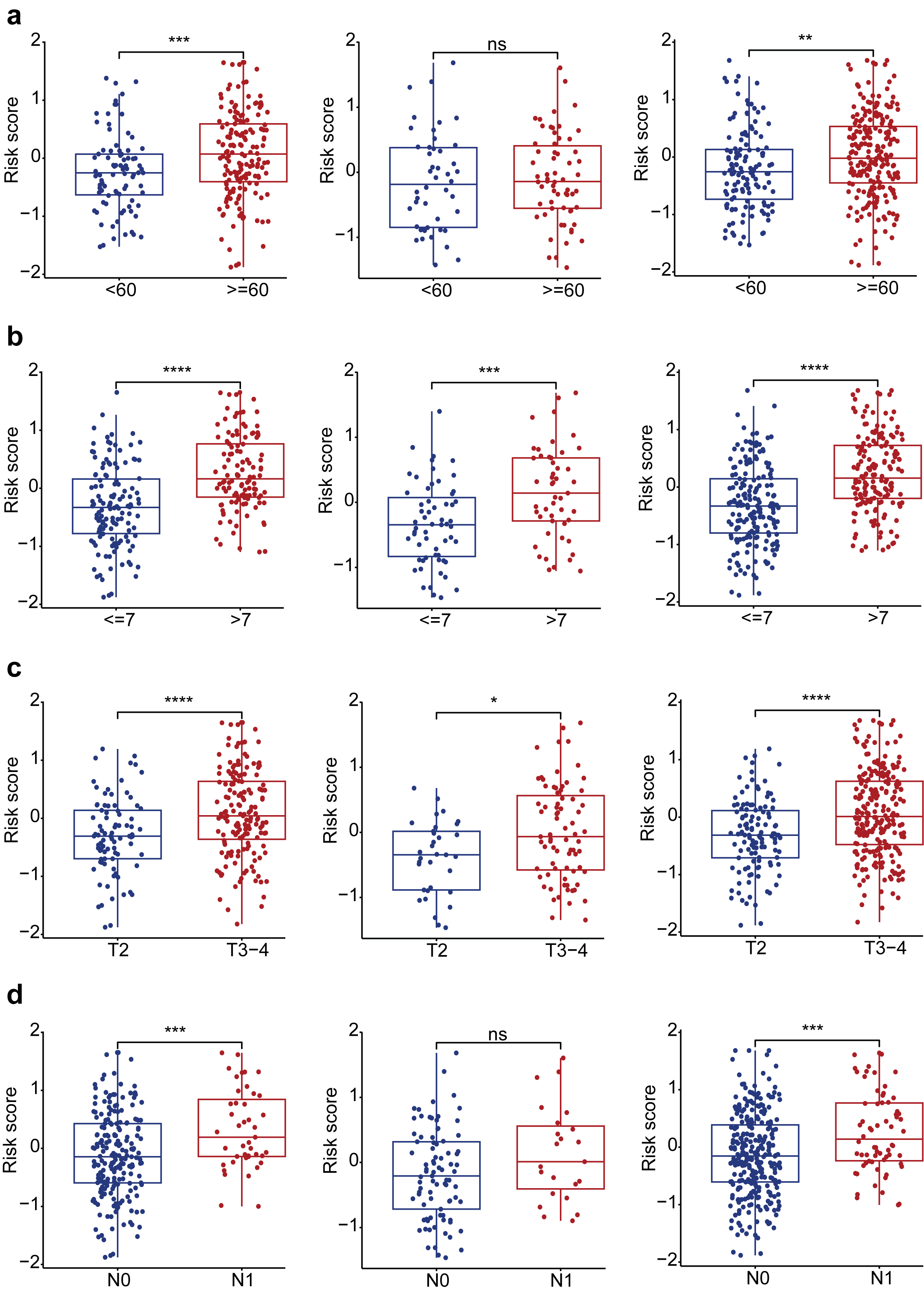

Supplement: Supplementary file 13 — Additional file 13: Figure S5. Clinical impact of the splicing event signature. Box plots of the alternative splicing event-based risk score in relation to (a) patient age at diagnosis (< 60 vs. ≥ 60), (b) Gleason score (≤ 7 vs. > 7), (c) pathological T stage (T2 vs. T3-T4), and (d) pathological N stage (N0 vs. N1). These plots are provided for the TCGA-PRAD training set (left panel), the testing set (middle panel) and the complete set (right panel). The significance of the risk score difference between the two groups is denoted with asterisks (ns, no significance, * p < 0.05, ** p < 0.01, *** p < 0.001, **** p < 0.0001). Pathological T stage: tumour stage; Pathological N stage: lymph node status (N0 = without lymph node metastasis; N1 = with lymph node metastasis). [file 40246_2023_545_MOESM13_ESM.tif]

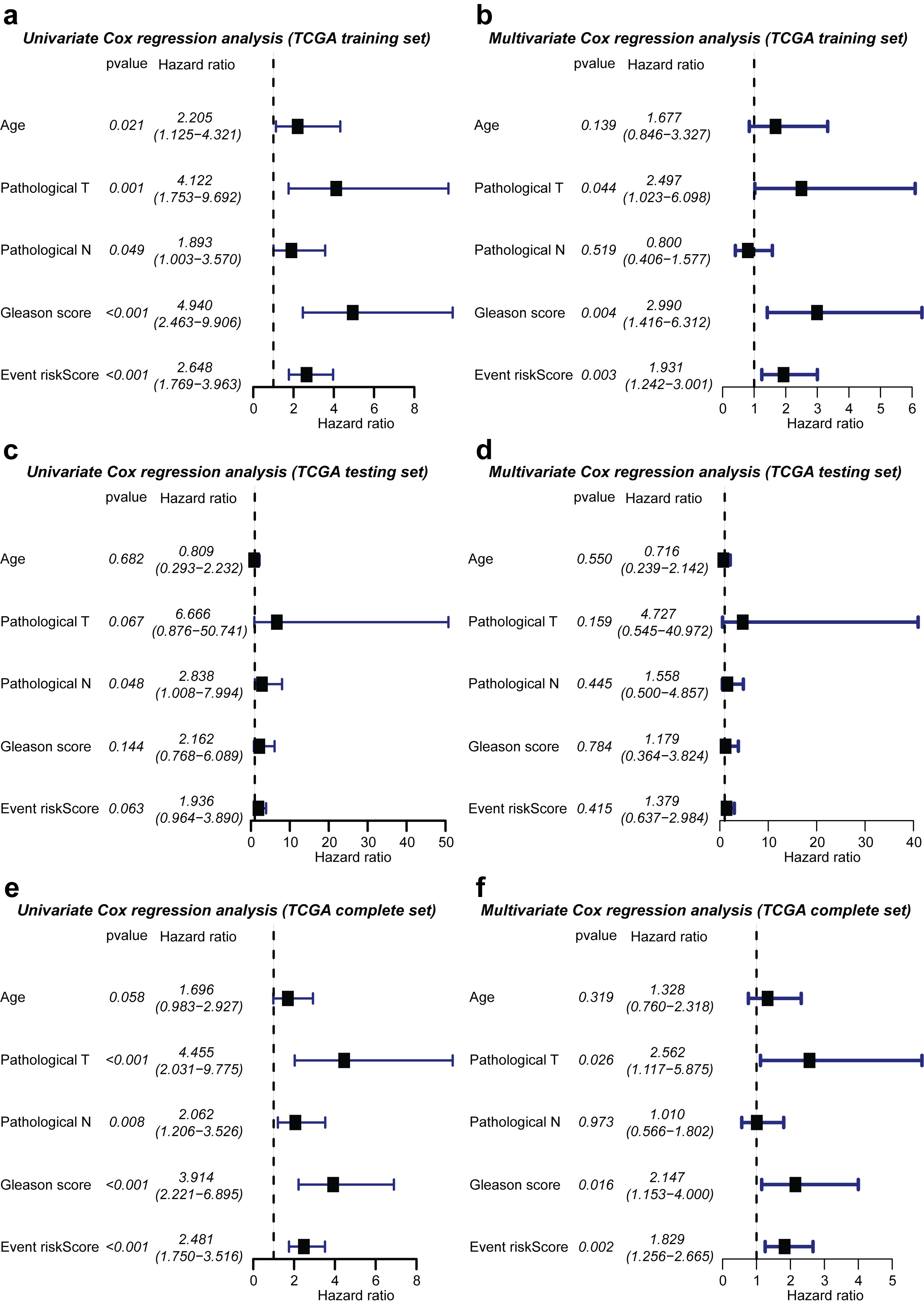

Supplement: Supplementary file 14 — Additional file 14: Figure S6. Assessment of the potential independent prognostic factors. Univariate and multivariate Cox regression analyses of alternative splicing event-based signature risk score and various clinicopathological variables, including age of the patient at diagnosis with prostate cancer (< 60 vs. ≥ 60), pathological T stage (tumour stage; T2 vs. T3-T4), pathological N stage (Lymph node status; N0 vs. N1) and Gleason score (≤ 7 vs. > 7). Forest plots of the respective univariate Cox and multivariate Cox in the TCGA-PRAD training set (a, b), the testing set (c, d), and the complete set (e, f). TCGA: The Cancer Genome Atlas; PRAD: prostate adenocarcinoma. [file 40246_2023_545_MOESM14_ESM.tif]

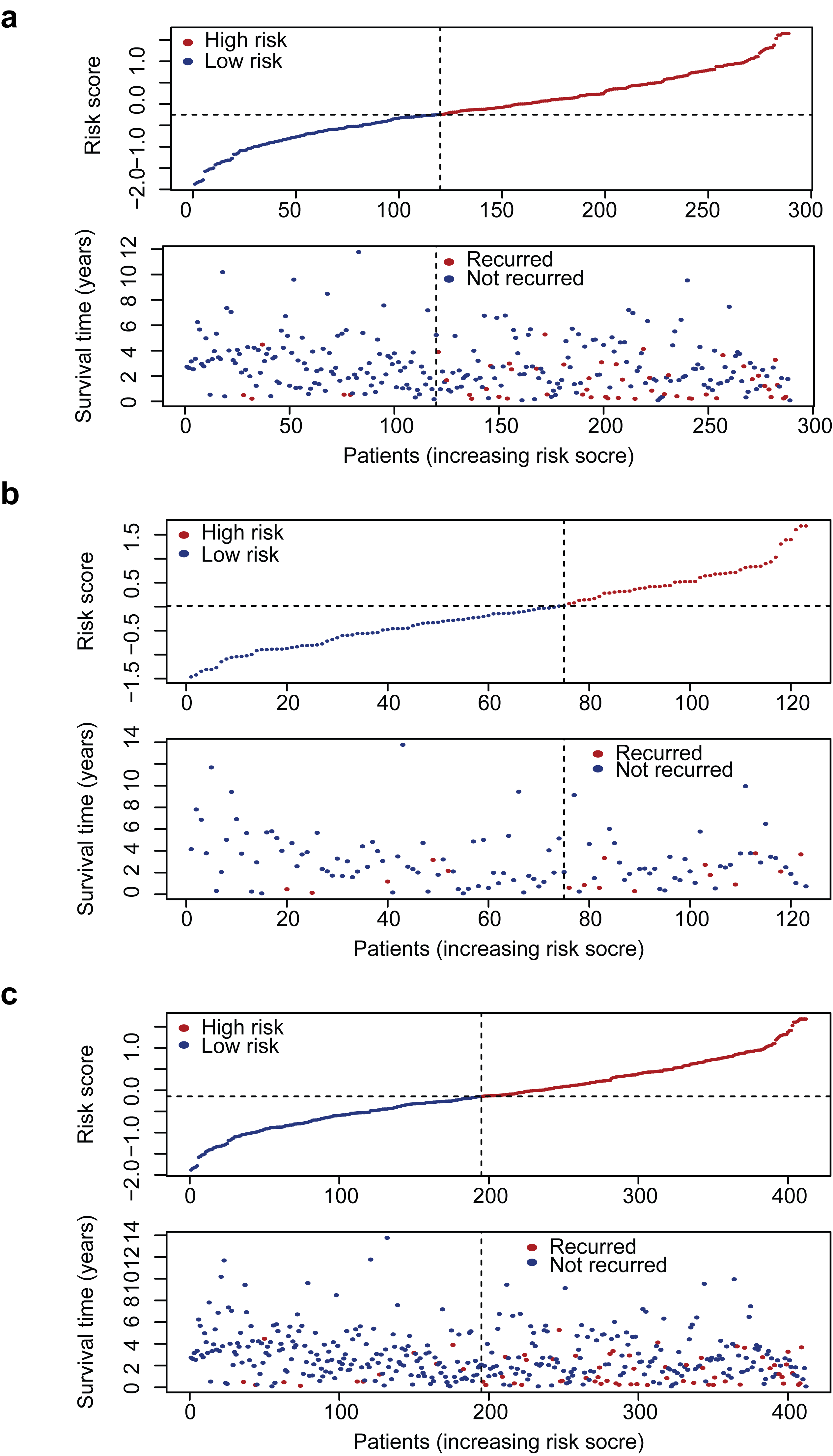

Supplement: Supplementary file 15 — Additional file 15: Figure S7. Risk score distribution (upper) and biochemical recurrence classification (recurred or not) in PCa patients (lower).These classifications are presented for the TCGA-PRAD training set (a), the testing set (b), and the complete set (c). PCa: prostate cancer; TCGA: The Cancer Genome Atlas; PRAD: prostate adenocarcinoma. [file 40246_2023_545_MOESM15_ESM.tif]
